# Approaching therapy of Alzheimer’s disease via the antidiabetic drug liraglutide—a study with streptozotocin intracerebroventricularly treated Wistar rats

**DOI:** 10.1007/s00702-025-02979-z

**Published:** 2025-07-12

**Authors:** Ana Knezovic, Michael Hosch, Catharina Sophia Hamann, Sandy Popp, Gabriela Ortega, Jelena Osmanovic-Barilar, Edna Grünblatt, Camelia Monoranu, Peter Riederer, Melita Salkovic-Petrisic, Angelika Schmitt-Böhrer

**Affiliations:** 1 Department of Pharmacology and Croatian Institute for Brain Research, University of Zagreb School of Medicine, Zagreb, Croatia; 2 Center of Mental Health, Department of Psychiatry, Psychosomatics, and Psychotherapy, University Hospital of Würzburg, Würzburg, Germany; 3 Department of Child and Adolescent Psychiatry and Psychotherapy, Psychiatric University Hospital Zurich, University of Zurich, Zurich, Switzerland; 4 Department of Neuropathology, Institute of Pathology, University of Wuerzburg, Würzburg, Germany

**Keywords:** Alzheimer’s disorder, Rat, Animal model, Streptozotocin intracerebroventricular treatment, GLP1-analogon, Liraglutide

## Abstract

Risk factors for developing dementia include type 2 diabetes and obesity. Streptozotocin (STZ) intracerebroventricularly (icv) treated rats are a well-accepted animal model for the sporadic Alzheimer`s disease (sAD). STZ-icv treatment results in an insulin-resistant brain state, cognitive deficits, and reduced hippocampal adult neurogenesis (AN). As the antidiabetic drug liraglutide (LIR) has been shown to improve AN, and spatial learning in an AD mouse model, we have investigated the effects of LIR treatment on spatial and fear-motivated learning, AN, and gene expression profiles in STZ-icv treated rats. Male Wistar rats were injected icv with STZ (f.c. 3 mg/kg) or vehicle. Two months later, four weeks of subcutaneous treatment with LIR (0.3 mg/kg) began. Cognitive abilities were assessed with the Morris water maze (MWM) and passive avoidance (PA) test. We performed quantitative immunohistochemistry to evaluate AN, and quantitative real-time PCR to determine the expression levels of genes involved in insulin signaling, glucose uptake, and neuroinflammation. STZ-icv rats showed significantly impaired spatial learning performance in the MWM and fear-motivated memory deficits in the PA test accompanied by reduced AN, downregulated insulin system- and glucose metabolism-related genes in the hippocampus and prefrontal cortex. LIR treatment did not reverse these cognitive deficits of STZ-icv rats in the MWM, and did even worsen PA performance. However, LIR partially restored dysregulated gene expression, however, additionally stimulated neuroinflammation. Refined experimental designs, e.g., refined dosing, should help to further clarify the therapeutic potential of LIR in the future.

**Supplementary Information:**

The online version contains supplementary material available at 10.1007/s00702-025-02979-z.

## Introduction

With a prevalence of about 50 million people worldwide (Georges et al. 2020; Tahami et al. 2024), dementia and Alzheimer’s disease (AD) are in charge of 1.3 trillion US dollars of costs globally each year and the seventh leading cause of death worldwide (WHO). The prevalence is expected to triple by 2050 (Patterson 2018). AD is the most common neurodegenerative disorder in Europe (Deuschl et al. 2020). Apart from advanced age (> 65 years) and carrying one or two APOE ε4 alleles being the strongest risk factors (van der Lee et al. 2018b, a), type 2 diabetes (T2DM), obesity, and mild cognitive impairment (MCI) are well accepted risk factors for developing dementia and especially the sporadic form of AD (sAD) (Livingston et al. 2024) accounting for over 95% of AD cases (Masters et al. 2015). In contrast, early onset or familial AD caused by mutations in genes (e.g. Presenilin 1 & 2, amyloid precursor protein) as hereditary factors is rare and usually manifests by age 60 (Wu et al. 2012).

In general, patients with AD suffer from memory loss, cognitive decline, impaired spatial navigation, and many more (Bondi et al. 2017). These symptoms are accompanied by atrophy of the hippocampus and cerebral cortex but also of subcortical structures such as the amygdala (Wenk 2003; Pini et al. 2016). Furthermore, neuroinflammation, a key hallmark of neurodegenerative diseases, is a prominent and early feature of AD, involving the activation of glial cells such as microglia and astrocytes (Ransohoff 2016).

MCI as a prodromal form of AD caught the eye of scientists (Bondi et al. 2017) and by that correlating risk factors for sAD, like T2DM (Dineley et al. 2011). The important role of insulin in energy homeostasis, memory enhancement and its effect on brain neurotransmitters leads to the hypothesis of AD being a “Type 3 diabetes” (de la Monte & Wands 2005; Janoutová et al. 2022; Michailidis et al. 2022). Concurrent with this notion, higher plasma insulin levels have been found in sAD patients (Watson et al. 2003), as well as a decrease in density of the insulin receptor (IR) (Frölich et al. 1999). Neurodegenerative processes seen in sAD are hypothesized to be driven by a combination of disturbances in insulin signaling as well as glucose uptake and energy metabolism, leading to a form of insulin-resistant brain state (IRBS) (Cardoso et al. 2009; Correia et al. 2011; Salkovic-Petrisic et al. 2009; Talbot et al. 2012). The classic cellular features of AD are shown to be more pronounced in patients with both AD and diabetes, suggesting that AD pathology in these patients is more severe and the progression of the disease is faster (Valente et al. 2010).

Most of the animal models used to study AD are based on the less frequent inherited AD (Chen and Zhang 2022). However, the intracerebroventricular (icv) treatment with the betacytotoxic drug streptozotocin (STZ) provides a well-accepted animal model for sAD (Salkovic-Petrisic and Hoyer 2007, Salkovic-Petrisic et al. 2013; Grieb 2016). STZ-icv treated animals (mostly rats and mice) share many features with sAD in humans (for review: Salkovic-Petrisic and Hoyer 2007, Salkovic-Petrisic et al. 2013; Kamat et al. 2016); IRBS associated with cognitive deficits (e.g. impaired spatial learning), progressive cholinergic deficits (Blokland and Jolles 1993; Bavarsad et al. 2020), glucose hypometabolism, oxidative stress, neuroinflammation and neurodegeneration (e.g. cerebral amyloid plaques or tau neuro-/angiopathy; Grünblatt et al. 2007; Salkovic-Petrisic et al. 2009, 2011; Knezovic et al. 2015; Li et al. 2020a). Additionally, adult neurogenesis (AN) was shown to be reduced in the hippocampus after STZ treatment in vivo (Sun et al. 2015) and in vitro (Sun et al. 2018).

As a causal relationship between diabetes and sAD becomes increasingly likely, antidiabetic drugs appear to be promising drugs to target sAD prevention and/or sAD treatment. A new class of antidiabetic drugs, the glucagon-like peptide-1 (GLP-1) receptor agonists such as exenatide, liraglutide (LIR), lixisenatide, and dulaglutide, are not only used therapeutically in diabetes patients, but some of them have already been shown to have powerful protective effects on cognitive function and can reverse or prevent neurodegeneration in animal models for AD (STZ-icv models: Xiong et al. 2013; Palleria et al. 2017; Zhou et al. 2019; the amyloid-ß-oligomers model: Batista et al. 2018; for review: Monti et al. 2022). The incretin hormone GLP-1 itself, but also some of its analogues, exert neuroprotective effects and prevent cognitive deficits in STZ-icv treated rats (Li et al. 2012, 2020b). Moreover, studies have demonstrated that LIR promotes AN in a genetic mouse model for AD (McClean et al. 2011; Parthsarathy and Hölscher 2013). In the present study, we aimed to investigate the effects of LIR treatment on spatial learning and AN, as well as its influence on expression profiles of genes related to the insulin system, glucose metabolism, and neuroinflammation in rats three months after STZ-icv injections.

## Material and methods

### Animals

A total of 32 four-month-old male Wistar rats were used for this study (Department of Pharmacology, University of Zagreb School of Medicine). The rats were kept 2–3 per cage in a controlled environment (temperature: 21–23 °C, relative humidity: 40–70%) with a 12 h light/12 h dark cycle (lights on 7 a.m.–7 p.m.). Standard food pellets and water were provided ad libitum. Body weight was measured every other day throughout the experiment.

### Ethics statement

All animal procedures were carried out in compliance with current institutional (University of Zagreb School of Medicine), national (Animal Protection Act, NN 102/17) and international (Directive, 2010/63/EU) guidelines on the use of experimental animals. The experiments were approved by the Ethical Committee of the University of Zagreb School of Medicine and the national regulatory body responsible for issuing ethical approval, Croatian Ministry of Agriculture (Licence No. or Registry number: 380-59-1010616-20/98; Classification: 641-01/16-02/01) for research approved by Croatian Ministry of Science, Education and Sport and Deutscher Akademischer Austauschdienst). 

#### Drug treatments

##### Intracerebroventricular injections

STZ-icv-injections were performed according to the procedure described first by Noble et al. [Noble et al. 1967] and applied afterwards by Salkovic-Petrisic and co-workers [Salkovic et al. 1995; Grünblatt et al. 2007; Salkovic-Petrisic et al. 2011, 2013; Sun et al. 2015]. Briefly, rats were given general anesthesia [chloralhydrate, 300 mg/kg intraperitoneally (i.p.), and STZ (1.5 mg/kg, dissolved in 0.05 M citrate buffer pH 4.5) was injected into the left and right lateral ventricle (2 μL per ventricle) of the rat brain. STZ treatment was repeated on the 3rd day after the first injection. Control animals received an equal volume of vehicle (citrate buffer) icv. The STZ dose was selected based on our previously published study, in which we investigated cognitive, structural, and ultrastructural alterations in the brain of the STZ-icv rat model over a 9-month period using three different doses. Among these, the 3 mg/kg dose produced the most pronounced effects on cognitive impairment and neuropathological hallmarks, including amyloid-β accumulation and hyperphosphorylated tau protein (Knezovic et al. 2015).

##### Liraglutide treatment

Two months after icv injections, half of the rats in each icv-treatment group (either vehicle-icv-treated or STZ-icv-treated) were subcutaneously (s.c.) injected with LIR (0.3 mg/kg) or saline (SAL) once daily for 4 consecutive weeks.

#### Experimental design

**Fig. 1:**
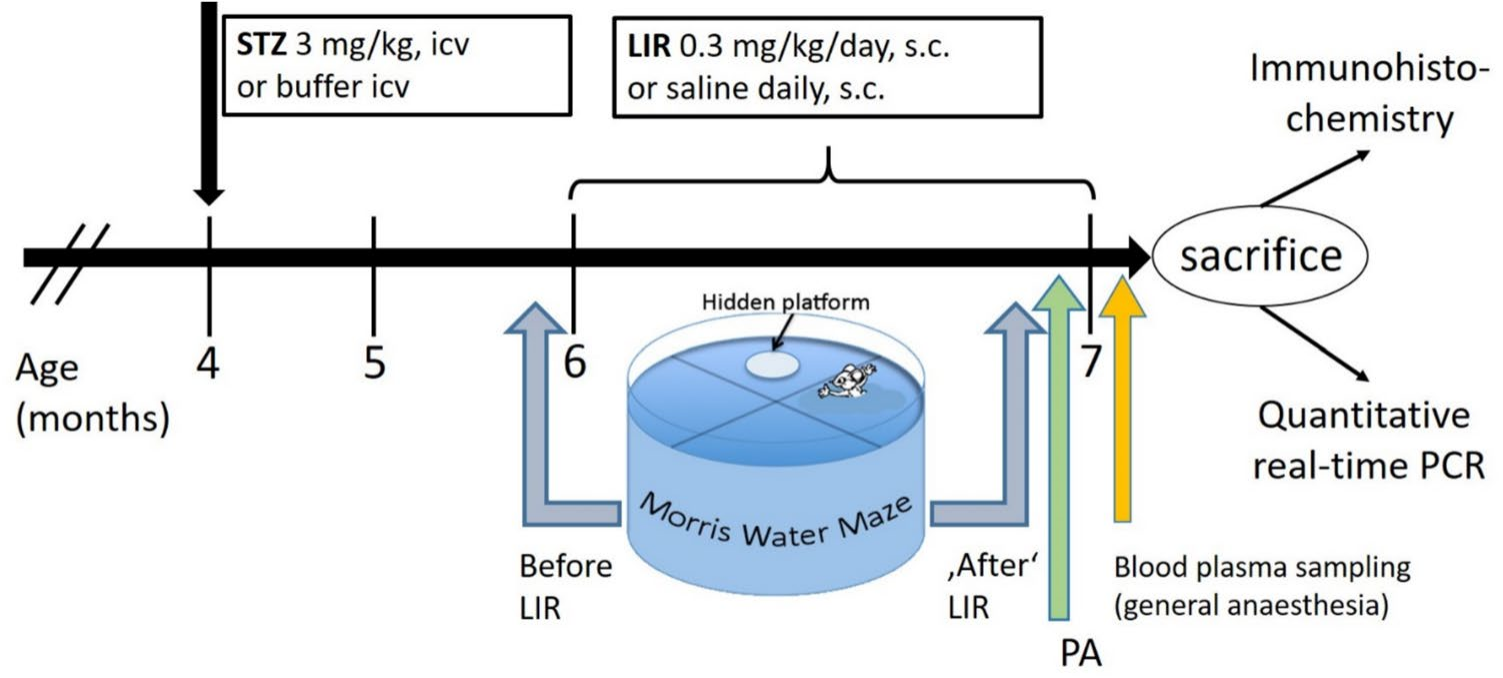
Timeline of the experiment. Streptozotocin (STZ) intracerebroventricular (icv) injections (twice) were performed in rats at four months of age. Subcutaneous (s.c.) treatment with Liraglutide (LIR) started 2 months after STZ-icv treatment for 1 month. Blood plasma samples were taken prior to sacrifice under general anesthesia. Behavioral tests: Morris water maze (MWM) before and in the fourth (final) week of LIR treatment (pre-test and post-test) with subsequent Passive avoidance test (PA)

Experimental rats were randomly divided into four experimental groups with 8 animals per group: Group 1 (VEH/SAL = vehicle (VEH) icv-treated rats, with Saline (SAL) s.c. treatment; n = 8); Group 2 (VEH/LIR =, vehicle icv-treated rats plus LIR s.c. treatment; n = 8); Group 3 (STZ/SAL = STZ-icv-treated rats with SAL s.c. treatment; n = 8) Group 4 (STZ/LIR = STZ-icv-treated rats plus LIR s.c. treatment; n = 8) (see Fig. 1). Therefore, with groups 1 to 4 we choose a 2 × 2 design. Two STZ-icv treated rats died in the last few weeks of the experiment, and one rat of the STZ/SAL group behaved completely abnormally. All three rats were therefore excluded from the analysis. Irrespective of treatment, all surviving animals were sacrificed at the age of 7 months.

#### Cognitive testing

Cognitive functions were assessed using the Morris water maze (MWM) and passive avoidance (PA) test. MWM was performed twice, just before the onset of LIR treatment and again during days 1 to 6 of the fourth (final) week of LIR s.c. treatment. First MWM test was performed with the aim of equalizing the SAL/LIR treatment groups based on their cognitive capability. The second MWM test was followed by the step-through PA test during days 6 to 8 of the final week with LIR treatment and ended one day before sacrifice.

##### MWM Swimming Test

The procedure of MWM [Morris 1984; Vorhees and Williams 2006] consisted of 5-day learning and memory training trials, and a probe trial on day 6, all performed in a 180 cm-diameter round pool, 60 cm deep, with water temperature set at 25 ± 1 °C. On days 1–5, the rats were trained to escape the water by finding a hidden glass platform (15 cm diameter, submerged 2 cm below the water surface) at a fixed position (at the edge of the NorthWest/NW quadrant). Staying on the platform in order to memorize its location was allowed for 15 s. Four consecutive trials were performed per day, each from a different starting position (SouthWest/SW, SouthEast/SE and NorthEast/NE), separated by a 30-min rest period (intertrial interval, ITI). The rats were released into the pool facing its wall. There were three visible visual cues positioned outside the pool on the walls. The platform was kept at the same position during the training period, but the starting position schedule changed.

On day 6, a probe trial which tested memory retention was performed (from quadrant SouthEast/SE) with the platform removed from the pool, where the time spent searching for the platform within the NW quadrant was recorded.

The cut-off time for each trial (training sessions as well the probe trial session) was set to 60 s. The escape latency (time needed to find the platform after being released into the pool) was recorded during all training trials. In addition, the number of search errors (entries into non-target quadrants¸ i.e. quadrants other than NW where the platform had been located) and total swim distance was recorded during all training trials as well as during probe trials. Time spent in target (NW) and non-target (NE, SW, SE) quadrants was measured during probe trial. Data were recorded by camera (Basler AG) and tracked and analyzed using EthoVision XT video tracking software (Noldus Information Technology).

##### PA

PA behavior was studied with a step-through type PA test utilizing the natural preference of rats for dark environments [Walters and Abel 1971]. The PA test is a fear-motivated avoidance task where rats learn to avoid stepping through a door to an apparently safer but previously punished dark compartment. The latency to avoid crossing into the punishment compartment serves as an index of the ability to avoid and allows memory to be evaluated. The apparatus used was an automated conditioning chamber from Ugo Basile (Comerio, Italy) divided into a brightly (with 22,000 Lux) illuminated “light” and a non-illuminated “dark” compartment (Chamber dimensions: 22.5 × 21 × 19 (height) cm each) by a partition containing a sliding door and equipped with a metal grid floor to deliver electric foot shocks.

The PA training procedure comprised 3 steps, each separated by 24 h and with a cut-off time of 5 min. The PA test performance started with a *habituation day* (day 1). Rats were allowed to freely explore to familiarize them with the novel environment, they did not receive a foot shock and the time needed to enter the dark compartment (*pre-shock latency*) was recorded. On subsequent day (day 2, the *conditioning* day), rats were placed in the light compartment and as soon as respective rat enters the dark compartment, the door was closed and the rat received a mild electric foot shock (0.3–0.5 mA depending on animal’s weight, 2 s in duration). Again, the step-through latency was recorded. And lastly, during the third testing day (*retention* day) rats were returned to the conditioning chamber and the time they needed to enter the previously punished dark compartment (post-shock latency) was recorded as an index of memory retention (without foot shock).

#### Tissue/blood sampling

Following behavioral testing (see above, Fig. 1), terminal blood samples were collected under anesthesia (thiopental 50 mg/kg/diazepam 5 mg/kg i.p.) immediately prior to sacrifice followed by a quick dissection of the brain. One hemisphere was used for quantitative immunohistochemistry to evaluate hippocampal AN. For that, respective hemisphere was fixed by immersion in 4% paraformaldehyde (PFA, dissolved in PBS, pH 7.5) for 72 h and further processed as described below. The other hemisphere as well as the hindbrain together with the cerebellum was immediately frozen in precooled isopentane for later use in a quantitative real-time PCR study. All brain tissues were then transported from the Department of Pharmacology and Croatian Institute for Brain Research (University of Zagreb School of Medicine, Zagreb, Croatia) to the Department of Psychiatry, Psychosomatics and Psychotherapy (University Hospital of Würzburg, Würzburg, Germany). After their arrival at the Department of Psychiatry, Psychosomatics and Psychotherapy in Würzburg, hemispheres, which had been fixed in 4% PFA, were then transferred to 10 and 20% sucrose in PBS. Subsequently, these hemispheres were frozen in precooled isopentane and stored at − 80 °C. Serial coronal sections were cut at 50 μm on a freezing microtome. These free-floating sections were collected in a one-in-six series, placed in 24-well plates each well filled with 1×TBS (1 ml).

### Biochemical analyses

#### Blood glucose concentration analysis

Withdrawal of blood samples taken from the tail vein of not fasting rats were carried out in deeply anaesthetized animals. Blood was sampled into tubes with heparin and centrifuged for 10 min at 3000 rpm at 4 °C. The supernatant (plasma) was collected. Blood glucose concentration was measured spectrophotometrically (by the method first described by Trinder (1969) using a commercial kit (Glucose GOD-PAP, Greiner DIAGNOSTIC GmbH). The measurement was conducted in strict compliance with the manufacturer’s protocol.

#### Quantitative real-time PCR study

Four brain regions (hippocampus, prefrontal cortex, hypothalamus and caudate putamen) were dissected from the fresh frozen hemisphere. Either the left or the right hemisphere were randomly chosen for brain region dissection. RNA extraction was performed by using miRNeasy Mini Kit (QIAGEN) as recommended by the manufacturer. After a quality check of the obtained total RNA using the NanoDrop® ND-1000 Spectrophotometer 1000 ng of each sample was used for cDNA synthesis which was performed using the iScript™ kit (Bio-Rad, Munich, Germany). Quantitative real-time PCR (qPCR) was performed employing the Bio-Rad CFX384 Real-Time PCR Detection System in technical triplicates. PCR was performed in 384-well plates (Life technology, Gaithersburg, USA) using the following conditions: 95 °C, 2 min; 45 cycles of 95 °C for 5 s (denaturation) and 60 °C for 30 s (annealing and extension), and a final step at 95 °C for 10 s. Melting curve analysis performed after the PCR run confirmed that only one amplicon had been generated. Mean PCR efficiencies were calculated by LinReg (Ramakers et al. 2003). The following reference genes for normalization were tested for stability using QBase: cyclophilin A (*CycA, Ppia*), hypoxanthine phosphoribosyl-transferase 1 (*Hprt1*), ribosomal protein L13A (*Rpl13A*) and tyrosine 3-monooxygenase/tryptophan 5-monooxygenase activation protein, zeta (*Ywhaz*). Information about all primers used in this qPCR study is provided in supplementary Table 1 (Online resource 1). Relative expression data were calculated with the normalization factors obtained from QBase and the mean efficiencies from LinReg.

#### Immunohistochemistry

*Immunostaining for light microscopy.* Immunodetection of Double cortin (DCX), a marker of adult neurogenesis, and of minichromosome maintenance complex component 2 (MCM2), a marker of proliferating cells), in coronal brain sections was performed according to the procedure described by Karabeg et al. 2013 with the Avidin–Biotin-Complex (ABC) method with some minor changes. For the detection of DCX antigen retrieval was performed with 10 mM sodium citrate buffer (pH 8.5) for 35 min at 80 °C. The primary antibodies used were polyclonal anti-DCX antibodies (1:1000) (made in goat; Santa Cruz Biotechnology, Santa Cruz, CA, USA; sc-8066), and the polyclonal anti-MCM2 antibodies (1:500) (made in goat; Santa Cruz Biotechnology, Santa Cruz, CA, USA; sc-9839). Subsequently, for DCX and MCM2 immunohistochemistry sections were incubated with biotinylated anti-goat IgGs made in horse. Secondary antibodies were provided from Vector Laboratories (Burlingame, CA, USA) and applied in a 1:1000 dilution.

*Quantification of DCX and MCM2-immunolabeled cells*. An Olympus B×51 microscope (Olympus, Hamburg, Germany) coupled to the Neurolucida imaging system (Microbrightfield, Inc., Willsiton, VT, USA) was used to acquire representative images from the examined individuals and to quantify DCX- immunoreactive (ir) cells found in the granular cell layer (GCL) as well as in the subgranular zone (SGZ). The volume of the GCL in each brain was also assessed via the Neurolucida system. With the resulting area from each brain slice, the slice thickness and the number of series, we were able to extrapolate the GCL volume for each brain hemisphere. Labeled cells were counted in the hippocampus in every sixth 50 μm thick section. This resulted in an analysis of an average of 8 sections per rat brain. Experimenters blind to the treatment did all the assessments. Immunopositive cells were counted at 20 × 1600 magnifications using the serial section manager function of the Neurolucida software. For quantitative evaluation a fictive coronal separation plane along the septotemporal axis of the hippocampus was used to divide the data obtained from anterior and posterior sections. The first section that showed the corpus callosum disconnected inside the two hemispheres was declared as the first section of the temporal part of the hippocampus (Karabeg et al. 2013; Sun et al. 2015). All sections before that point were considered to hold the septal part of the hippocampus (between interaural 1.26 and 1.34 mm; [84]). Some sections were lost in the process of cutting or during free-floating immunohistochemistry, which resulted in an irregular number of sections. Therefore, those brain hemispheres that had less than three sections within the anterior or posterior part of the hippocampus were excluded from the evaluation of the anterior and posterior hippocampus, respectively. After this adjustment, the density of immune-positive cells of the SGZ/GCL was calculated per anterior or posterior hippocampus for each brain.

### Statistical analysis

Data were analyzed by two-way analysis of variance (ANOVA) with group (STZ vs. VEH) and treatment (LIR vs. SAL) as between-subjects factors or, when appropriate, by mixed-design ANOVA with additional repeated measures factors (i.e., treatment day for body weight, training day and phase [before and after treatment] for MWM). The Bonferroni’s multiple comparisons test or the uncorrected Fisher’s LSD post hoc test was used as a post hoc-test. Parametric assumptions were verified using Shapiro–Wilk normality test, Levene’s test for homogeneity of variances and Mauchly’s test of sphericity. The Greenhouse Geisser correction was applied if sphericity had been violated. The Kaplan–Meier method was used to describe time-to-event outcomes, i.e., the step-through latency in the PA test, and the log-rank test was used to analyze differences between the groups.

The Pearson correlation coefficient was performed to determine the relationship between DCX-ir cell densities in the anterior and posterior hippocampus and mean errors in the MWM.

## Results

### Body weight: Significant loss of body weight due to treatment with LIR

**Fig. 2:**
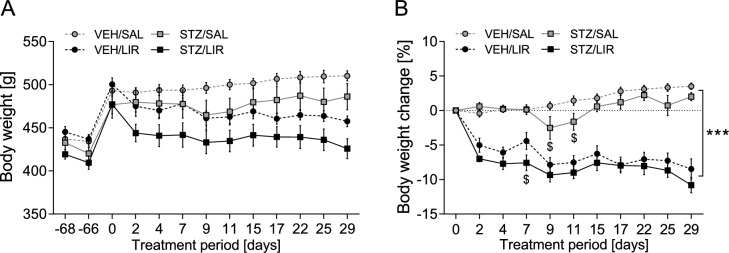
Time-course analysis of **A** absolute body weight and **B** relative weight change during the 4-week treatment period. Liraglutide (LIR) exerted rapid and long-lasting body weight loss in STZ-icv treated rats as well as VEH controls. Data are shown as mean ± SEM. ***p < 0.0001 for overall comparison of LIR-treated rats vs. SAL controls, ^$^*p* < 0.05 for comparison of STZ vs. VEH animals within SAL and LIR groups, respectively. A three-way mixed ANOVA including repeated measures (treatment day for body weight) with the Bonferroni’s multiple comparisons test as post hoc-test was applied

Three-way mixed ANOVA on body weight indicated that STZ-icv treated rats were uniformly leaner (~ 5%) than VEH controls throughout the entire study period (main effect of group: *F*_(1,25)_ = 6.419, *p* = 0.018; Fig. 2A). Besides, the analysis detected a significant main effect of time (*F*_(3.37,84.24)_ = 86.159, *p* < 0.0001) and treatment (*F*_(1,25)_ = 9.577, *p* = 0.005) along with a time × treatment interaction (*F*_(3.37, 84.24)_ = 25.169, *p* < 0.0001). Post hoc tests revealed a mild transient weight loss upon icv injection of either STZ or VEH (day − 68 vs. day − 66: *p* < 0.0001) followed by subsequent body weight gain of similar magnitude in control and experimental groups until treatment initiation (day − 66 vs. day 0: *p* < 0.0001; Fig. 2A).

After treatment onset, LIR-injected STZ and VEH rats rapidly lost weight within the first two days (*p* < 0.0001 compared to day 0) and continued to drop weight slowly and steadily thereafter (day 2 vs. day 29: *p* < 0.001; Fig. 2A). By contrast, SAL-injected animals gradually gained weight throughout the four-week treatment period (day 2 vs. day 29: *p* = 0.042; Fig. 2A). Accordingly, the difference in relative body weight between LIR- and SAL-treated rats became progressively larger over time and reached its maximum at the end of the treatment period (day 2: 6.10 ± 0.71%, day 29: 12.41 ± 1.04%, *p* < 0.0001; Fig. 2B). Moreover, a three-way mixed ANOVA on relative body weight change yielded a significant time × group × treatment interaction (*F*_(5.27,131.69)_ = 2.614, *p* = 0.025; Fig. 2B). Post hoc tests showed that the weight-reducing effect of LIR was slightly stronger in STZ-icv treated rats compared to VEH controls during the first week of treatment (day 7: *p* = 0.029) but indistinguishable between experimental groups thereafter (all *p* > 0.05). Furthermore, within the SAL-treated control group STZ-icv treated rats exhibited mild but significant weight loss relative to VEH animals on treatment days 9 (*p* = 0.049) and 11 (*p* = 0.033).

### Blood glucose: All blood glucose levels are within normal range

**Fig. 3:**
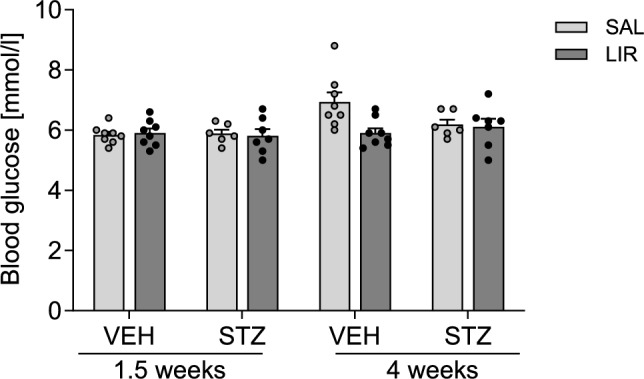
Blood glucose levels were determined at 1.5 and 4 weeks after LIR treatment onset. Data are shown as individual data points and additionally with mean ± SEM. A three-way mixed ANOVA test including repeated measures (day of measurement for blood glucose) was used

Postprandial blood glucose levels were assessed 1.5 weeks (day 10) and 4 weeks (day 29) after LIR treatment onset. Three-way mixed ANOVA yielded only a marginally significant time × group × treatment interaction (*F*_(1,25)_ = 3.781, *p* = 0.063; Fig. 3).

#### Morris Water Maze: STZ-icv-treatment induced significant impairment in spatial learning performance that could not be reversed by LIR treatment

##### Acquisition (spatial learning)

**Fig. 4:**
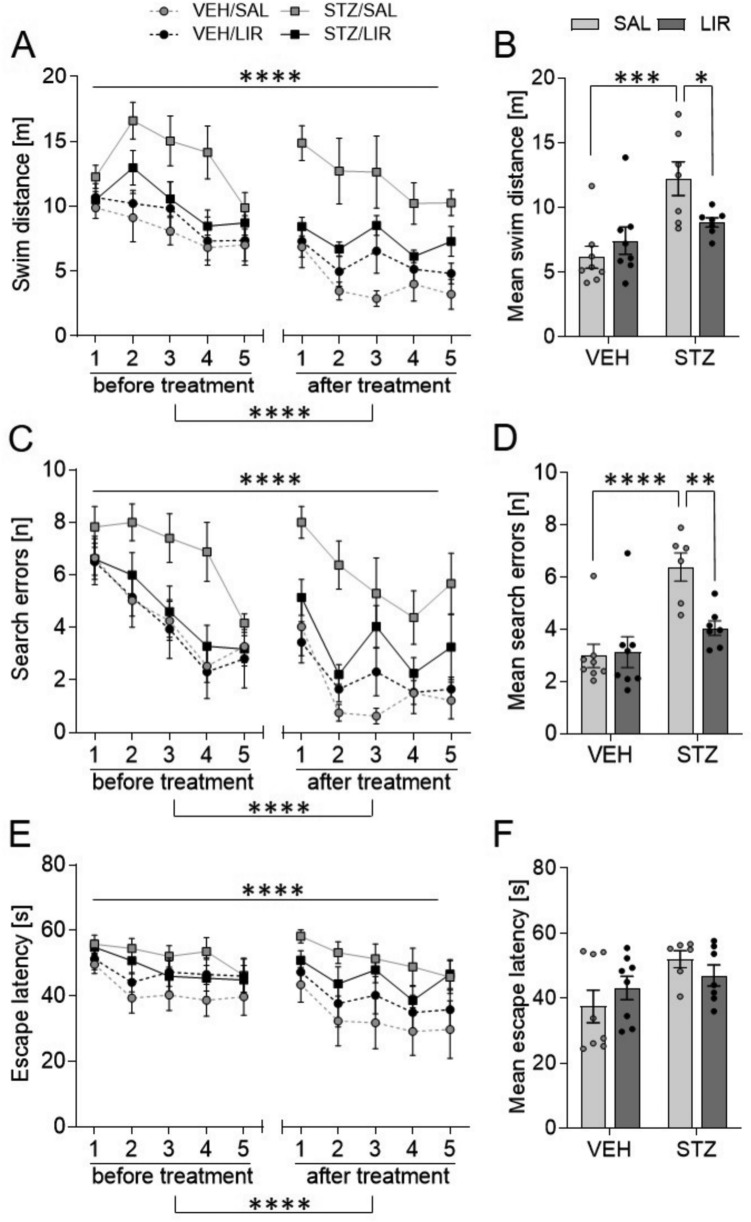
Rats were trained on five consecutive days once before (pretest) and after (posttest) LIR treatment to acquire spatial memory in the Morris water maze task. **A**, **C**, **E** Time-course analysis showing mean ± SEM values (averaged across four trials per day) for each training session. **B**, **D**, **F** Graphs show individual data points and mean ± SEM values (averaged across all training days before and after treatment. Parameters used to measure spatial learning performance included **A**, **B** total swim distance, **C**, **D** number of search errors (i.e., entries into non-target quadrants), and **E**, **F** escape latency (i.e., time to find the hidden platform). A four-way mixed ANOVA including repeated measures and the uncorrected Fisher’s LSD post hoc test were applied. ^*^p < 0.01, ^**^p < 0.01, ^***^p < 0.001, ^****^p < 0.0001

The main results obtained from four-way mixed ANOVA on behavioral measures in the MWM are summarized in Table 1. The analysis revealed significant main effects of phase (pretest vs. posttest) and training day on each dependent variable, indicating that swim distance (Fig. 4A), search errors (Fig. 4C) and escape latency (Fig. 4E) gradually decreased across sessions as the animals learned to locate the hidden platform. Furthermore, STZ-icv treated rats exhibited spatial learning deficits since they covered longer distances (Fig. 4A, B), made more mistakes (Fig. 4C,D) and required more time to reach the target (Fig. 4E, F) than VEH controls throughout all acquisition trials of the task (significant main effect of group on each variable). Besides the overall impaired performance of STZ animals, the difference between the two groups (STZ vs. VEH) became even more pronounced in the posttest (i.e., after LIR treatment) relative to pretest (i.e. before LIR treatment) scores due to steeper learning curves in VEH controls (Fig. 4A, C, E; phase × group interaction on each variable). Moreover, the analysis yielded a significant group × treatment interaction for swim distance and search errors, but not for escape latency. As the expected phase × group × treatment interaction was not statistically significant for any given variable, suggesting that detected group differences existed already before LIR treatment initiation, subsequent post hoc tests could only be performed using the mean of the pretest and posttest data. The applied uncorrected Fisher’s LSD post hoc test revealed that the spatial learning deficit was present in SAL-treated STZ-icv treated rats (p = 0.0001 vs. VEH/SAL for distance and *p* < 0.0001 vs. VEH/SAL for errors), which was attenuated in LIR-treated STZ-icv rats compared to SAL-treated STZ-icv treated rats (p < 0.05 for distance, and *p* < 0.01 for errors) (Fig. 4B, D).

**Table 1:** Summary of the four-way mixed ANOVA results on behavioral measures during acquisition of spatial learning in the Morris water maze test

Source of variation	*df*	Swim distance		Search errors		Escape latency	
		*F*-value	*p*	*F*-value	*p*	*F*-value	*p*
Within-subjects effects							
Phase	1	**66.936**	**0.000**	**48.385**	**0.000**	**8.773**	**0.007**
Phase × group	1	**4.860**	**0.037**	**6.032**	**0.021**	3.381	0.078
Phase × treatment	1	0.188	0.669	0.065	0.801	0.051	0.824
Phase × group × treatment	1	2.277	0.144	1.283	0.268	0.109	0.744
Error (phase)	25						
Day	4	**11.574**	**0.000**	**25.257**	**0.000**	**10.177**	**0.000**
Day × group	4	2.048	0.093	0.622	0.648	0.618	0.651
Day × treatment	4	0.871	0.484	0.474	0.755	0.542	0.705
Day × group × treatment	4	1.146	0.340	0.737	0.569	0.684	0.605
Error (day)	100						
Phase × day	4	**4.299**	**0.003**	**4.399**	**0.003**	0.751	0.559
Phase × day × group	4	1.377	0.247	1.662	0.165	0.513	0.727
Phase × day × treatment	4	1.146	0.340	1.346	0.258	0.206	0.934
Phase × day × group × treatment	4	0.479	0.751	0.666	0.617	0.281	0.890
Error (phase × day)	100						
Between-subjects effects							
Group	1	**18.213**	**0.000**	**19.553**	**0.000**	**5.478**	**0.028**
Treatment	1	2.082	0.161	5.021	**0.034**	0.009	0.926
Group × treatment	1	**7.799**	**0.010**	**6.421**	**0.018**	1.852	0.186
Error	25						

Bold letters indicate significant differences with *p* < 0.05

*Phase* pretest vs. posttest, *Group *STZ-icv vs. vehicle-icv, *Treatment* LIR vs. SAL

##### Probe trial (spatial reference memory)

**Fig. 5:**
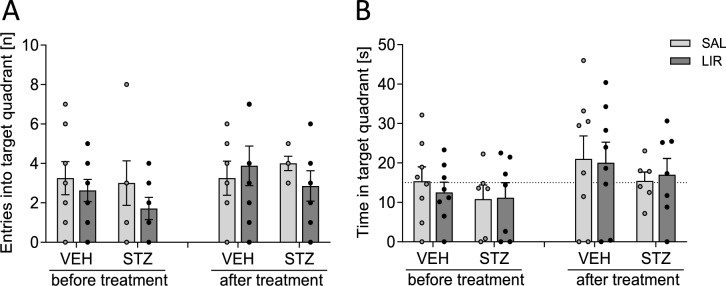
Rats were subjected to a probe trial with the platform being removed once before (pretest) and after (posttest) LIR treatment to determine spatial reference memory. Memory indices were **a** the number of entries and **b** the time spent in the target quadrant. A three-way mixed ANOVA test with repeated measures (phase: before and after treatment) was performed. Graphs show individual data points and mean ± SEM values. The dashed line in (b) indicates chance level

The main results obtained from three-way mixed ANOVA on behavioral measures in the MWM probe trials are summarized in Table 2. The analysis detected a significant main effect of phase on the number of target quadrant visits (Fig. 5A) and time spent in the target quadrant (Fig. 5B), indicating reference memory improvement in the posttest relative to pretest as the values increased from the 1 st to 2nd probe trial. However, spatial reference memory seemed to be comparable across control and experimental groups since neither of the relevant main nor interaction effects were statistically significant. Furthermore, one sample *t*-tests comparing the time each group spent in the target quadrant against chance level (test value = 15) indicated that neither control nor experimental groups showed a clear preference for the zone where the platform had previously been located.

**Table 2:** Summary of the three-way mixed ANOVA results on behavioral measures in the Morris water maze probe trials (reference memory tests)

Source of variation	*df*	Swim distance		TQ visits		TQ duration	
		*F*-value	*p*	*F*-value	*p*	*F*-value	*p*
Within-subjects effects							
Phase	1	2.375	0.136	**5.451**	**0.028**	**5.208**	**0.031**
Phase × group	1	0.001	0.976	0.377	0.544	0.069	0.795
Phase × treatment	1	0.031	0.862	0.919	0.347	0.091	0.766
Phase × group × treatment	1	0.919	0.347	0.580	0.453	0.004	0.949
Error (phase)	25						
Between-subjects effects							
Group	1	0.203	0.657	0.241	0.627	1.175	0.289
Treatment	1	0.720	0.404	0.698	0.411	0.019	0.893
Group × treatment	1	1.486	0.234	0.698	0.411	0.182	0.674
Error	25						

Bold letters indicate significant differences with *p* < 0.05

*TQ* target quadrant, *Phase* pretest vs. posttest

### Step-through passive (inhibitory) avoidance task: Both STZ and LIR treatments have negative effects on fear-motivated learning

In accordance with their exploratory drive and innate aversion to brightly illuminated areas, all rats quickly entered the dark compartment in the first two trials (habituation and conditioning, i.e., before receiving an electric shock). STZ-icv treated rats displayed slightly shorter transfer latencies than VEH controls in the habituation trial, but no difference between groups was detected in the conditioning trial (Online resource 2).

On day 3 (retention day), the animals with no alterations in memory functions (vehicle-icv treated, intact) were supposed to remember receiving a foot shock after entering the dark compartment and, therefore, to stay in the light compartment longer. The animals with damaged memory (STZ-icv treated) were supposed to have less memory of the received foot shock in the dark compartment, and thus to spend less time in the light compartment and enter the dark area more rapidly in comparison with the control rats.

**Fig. 6:**
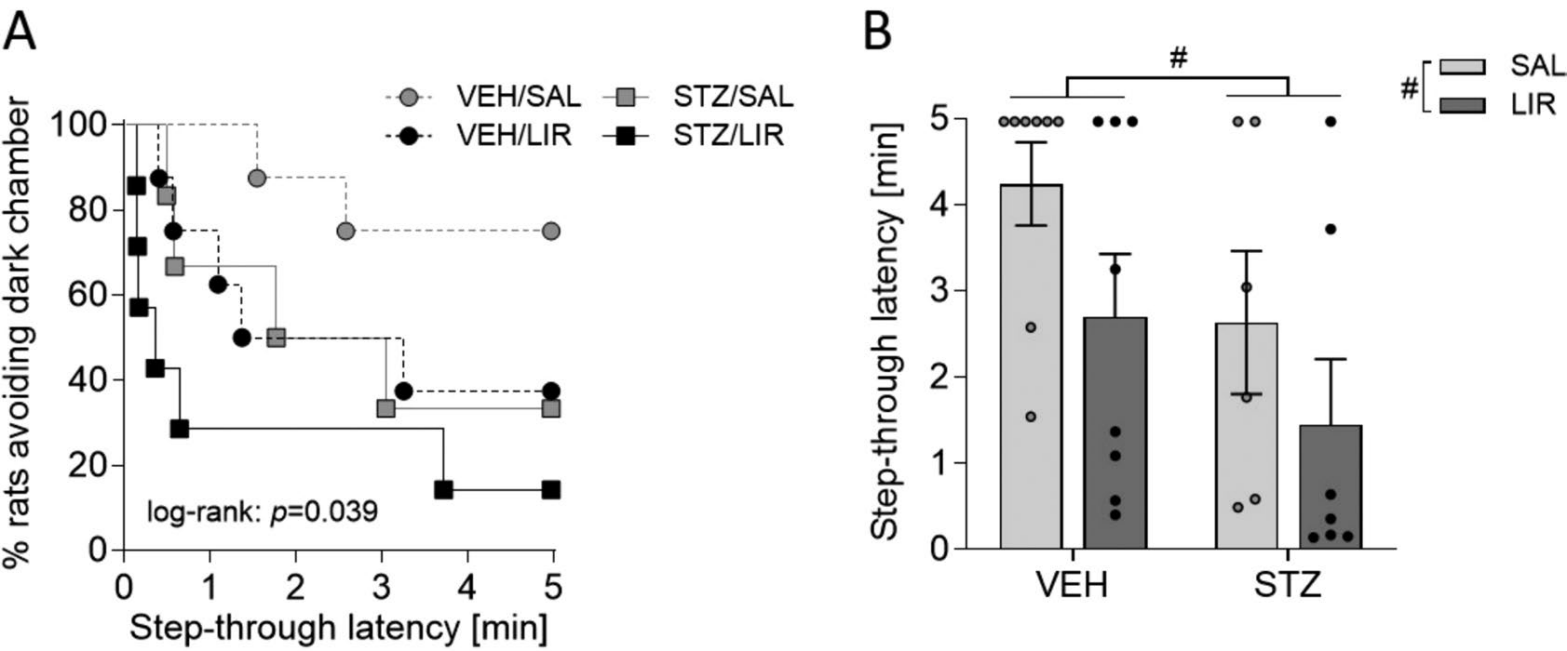
Fear-conditioned memory was assessed by passive avoidance test 24 h after conditioning. **A** Kaplan–Meier curves showing the percentage of rats that avoided the previously punished dark compartment during the 5-min retention test. The Kaplan–Meier method was used to describe time-to-event outcomes, and the log-rank test was used to analyze differences between the groups. **B** STZ-icv and liraglutide (LIR) treatment reduced the step-through latency in an additive manner. A two-way ANOVA test was performed. Data are shown as individual data points and mean ± SEM values with ^#^*p* < 0.1

Time-to-event analysis of the step-through latency in the 24 h memory retention test showed that 75.0% of VEH/SAL, 37.5% of VEH/LIR, 33.3% of STZ/SAL and 14.3% of STZ/LIR rats avoided the previously punished dark compartment (Fig. 6A). Comparison of the Kaplan–Meier curves revealed a statistically significant difference between the four treatment conditions (log-rank test: χ^2^(3) = 8.359, *p* = 0.039). Besides, a two-way ANOVA on the step-through latency revealed a marginally significant main effect of group (VEH vs. STZ: *F*_(1,25)_ = 4.124, *p* = 0.053) and treatment (SAL vs. LIR: *F*_(1,25)_ = 3.777, *p* = 0.063) without an interaction between the two factors (*F*_(1,25)_ = 0.064, *p* = 0.802), indicating that both STZ-icv and LIR treatment decreased the step-through latency and hence impaired fear memory retention in an additive manner (Fig. 6B).

### Adult hippocampal neurogenesis was reduced after STZ-icv treatment and remained unchanged after LIR treatment

**Fig. 7:**
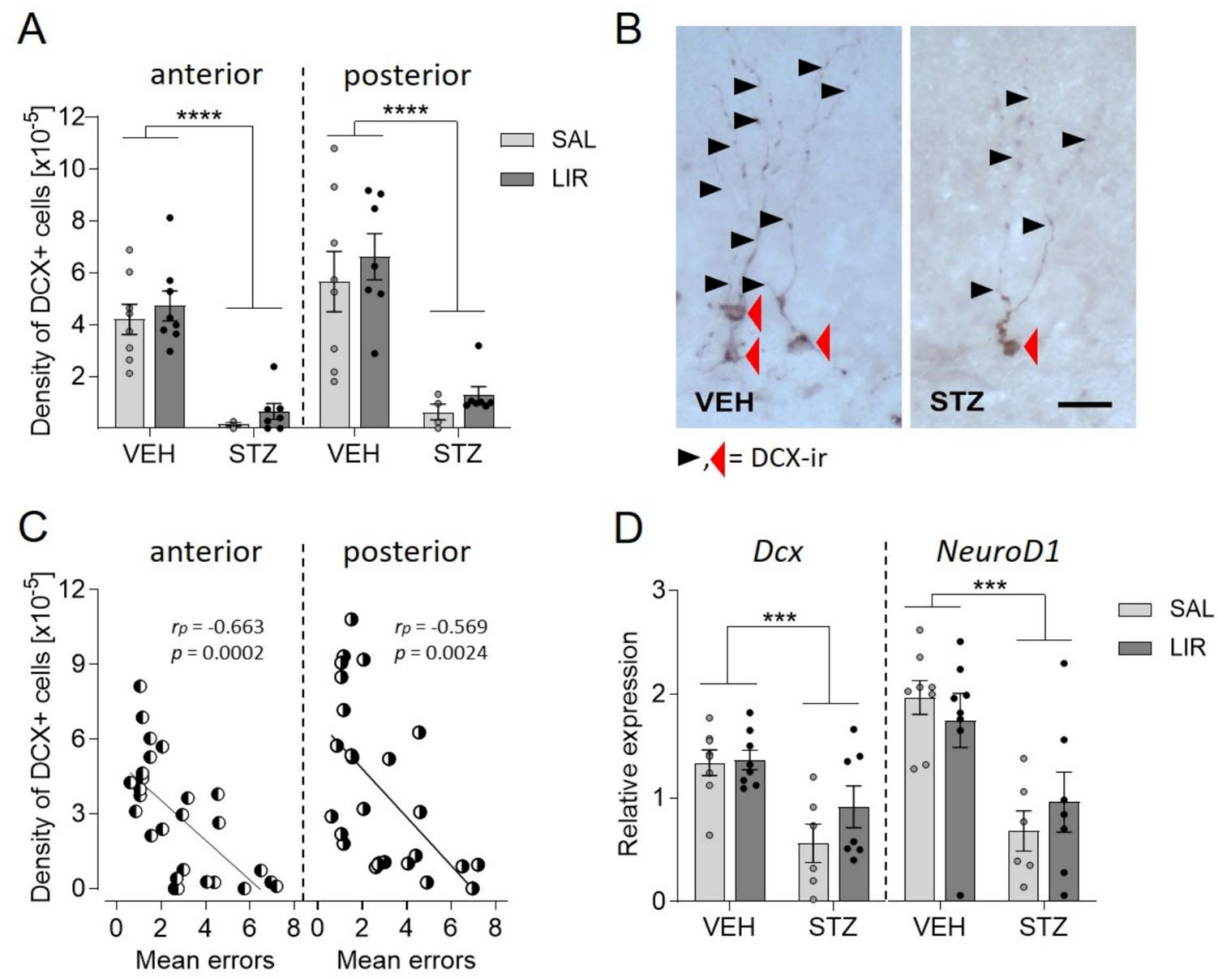
Adult hippocampal neurogenesis is strongly influenced by STZ-icv treatment, but not by LIR treatment. **A** Density of DCX-positive cells in the anterior and posterior hippocampus was strongly reduced in STZ-icv-treated rats. A two-way ANOVA was performed. **B** Representative images of DCX-immunoreactive (ir) cells in the SGZ of the Hippocampus of a VEH-treated and a STZ-icv-treated rat. Black arrowheads indicate DCX-positive dendrites, red arrowheads indicate DCX-ir cytosomata. **C** Density of DCX-positive cells correlated negatively with mean errors in the MWM. Data points represent DCX-ir cell density and mean errors. Correlations analyzed with the Pearson correlation coefficient are significant in anterior (n = 27) and posterior (n = 26) hippocampus. **D** Expression levels of the AN marker genes *Dcx* and *Neurod1* in the hippocampus confirm the negative effect of STZ treatment on AN as revealed by quantitative IHC. A two-way ANOVA was performed. Data are shown as individual data points and additionally mean ± SEM values with^***^p < 0.001, ^****^p < 0.0001

Key results obtained from two-way mixed ANOVA of gene expression data (mRNA level) and a quantitative immunohistochemical measure (protein level) dealing with the phenomenon of AN in the hippocampus are summarized in Tables 1 and 2 of online resource 3. The data obtained at both mRNA and protein levels revealed only significant main group effects (effects of STZ treatment), no main effect of LIR treatment and no interaction between groups and LIR treatment. Statistical analysis of the density of cells immunoreactive (ir) for DCX, a marker of newborn, but still immature neurons, in the anterior and posterior hippocampus indicate that STZ treatment diminished the birth of new neurons with high significance in both parts of the hippocampus [main effect of group in anterior hippocampus (*F*_(1,25)_ = 56.465, *p* < 0.0001) and posterior hippocampus (*F*_(1,25)_ = 31.234, *p* < 0.0001)] (Fig. 7A). Representative images of DCX-ir cells in the SGZ of the Hippocampus of a VEH-treated and a STZ-icv-treated rat are shown in Fig. 7B. Analyzing possible correlations between the density of DCX-positive cells and mean errors in the MWM resulted in a significant negative correlation (anterior hippocampus: rp(27) = − 0.663, p = 0.0002; posterior hippocampus with rp(26) = − 0.569, p = 0.0024; Fig. 7C). Quantification of an earlier stage of AN, the proliferation of progenitor cells, with antibodies detecting MCM2 did not reveal any differences between groups [no main effect of group, treatment and no interaction in the anterior hippocampus (*F*_(1,25)_ = 0.00, *p* = 1.0) and posterior hippocampus (*F*_(1,25)_ = 0.00, *p* = 1.0)] (not shown in Fig. 7). At the mRNA level statistical analysis of relative expression levels of *NeuroD1*, a bHLH transcription factor involved in neuronal differentiation, as well as *Dcx* indicate that STZ treatment diminished hippocampal AN also at gene expression level with high significance [main effect of group with *NeuroD1* (*F*_(1,25)_ = 19,496, p = 0.00017) and *Dcx*: (*F*_(1,25)_ = 16,496, p = 0,0004)] (Fig. 7D).

### Expression study of glucose metabolism- and insulin system-related genes: LIR treatment seems to partially restore the dysregulated gene expression profile of STZ injected rats.

Expression of several genes that play an important role in the insulin system, in glucose metabolism, and various relevant signaling pathways were investigated in prefrontal cortex (PFC), hippocampus, caudate putamen, and hypothalamus using qPCR. Results obtained from two-way mixed ANOVA with relative expression levels of all genes investigated such as *the glucose transporter (Glut)1, Glut 3, and Glut 4, insulin receptor (InsR), Insulin receptor substrate 1 (Irs1), Insulin growth factor 1 (Igf1), phosphatidylinositol-4,5-bisphosphate 3-kinase catalytic subunit alpha was (Pik3ca), ribosomal protein S6 kinase B1 (Rps6kb1; p70 S6K-alpha), glycogen synthase kinase 3 beta (Gsk3 b), and mechanistic target of rapamycin kinase (Mtor)* are summarized in Tables 1 and 2 of online resource 4. Results obtained from two-way mixed ANOVA on the relative expression data of almost all investigated genes examined revealed only significant main group effects (effects of STZ treatment), in no case a main effect of LIR treatment and only in few cases an interaction between groups and LIR treatment. Most gene expression differences between groups could be detected in PFC and Hippocampus.

**Fig. 8:**
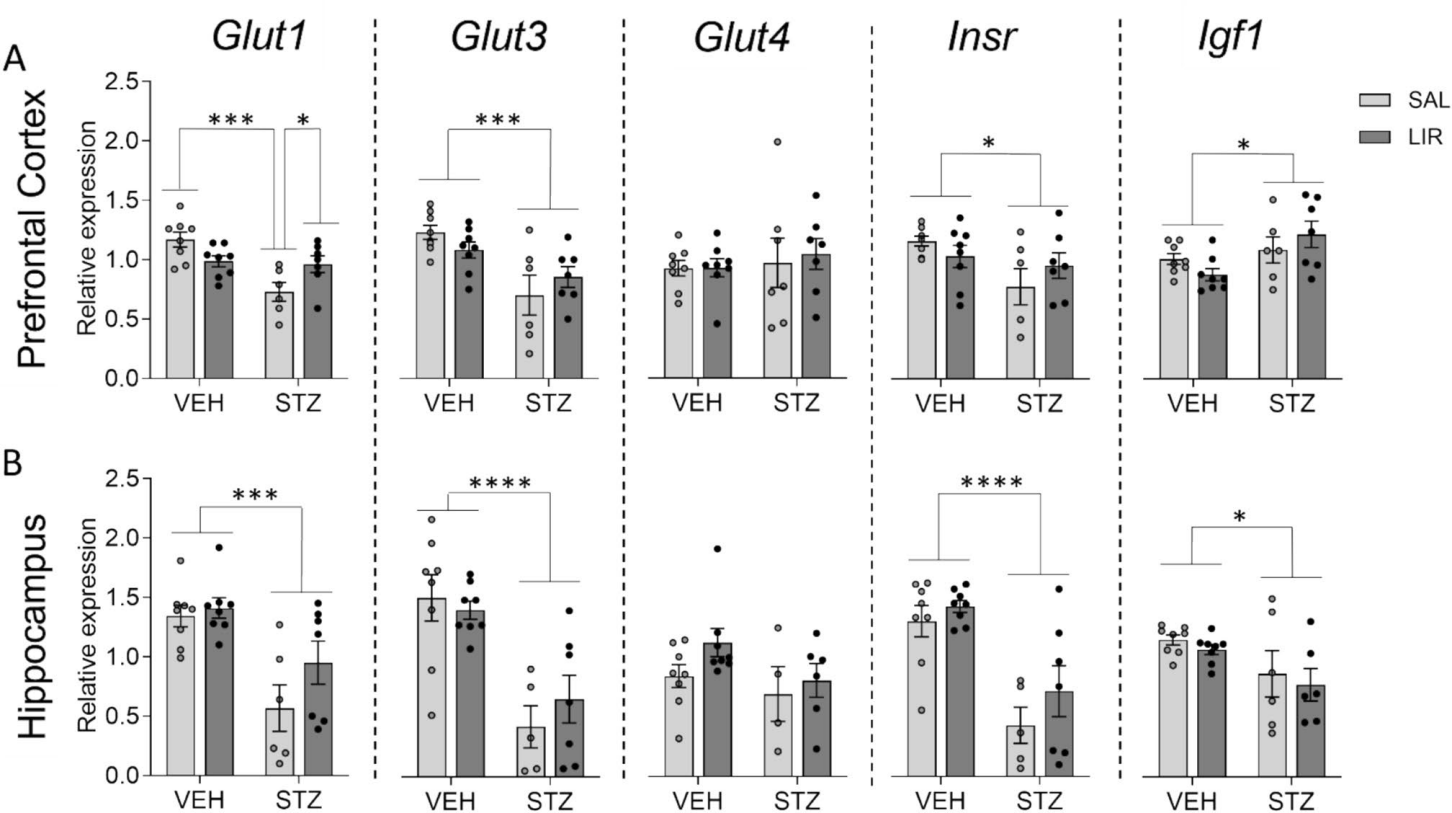
Expression levels of genes related to glucose metabolism and the insulin system such as glucose transporter (*Glut*) 1, 3 and 4, insulin receptor (*Insr*) and insulin-like growth factor (*Igf*)1 in the prefrontal cortex (**A**) and hippocampus (**B**). A two-way ANOVA test with Bonferroni’s multiple comparison test as a post hoc-test was applied. A significant group by treatment interaction could only be detected in the prefrontal cortex using primer pairs specific for *Glut1*. Data are shown as individual data points and mean ± SEM with ^*^p ≤ 0.05, ***p < 0.001, ****p < 0.0001

A selection of these gene expression results in PFC and Hippocampus is shown graphically in Fig. 8.

Exclusively in PFC with primer pairs specific for *Glut1* mRNA qPCR results show that LIR treatment seems to partially restore the dysregulated gene expression profile of STZ injected rats (Fig. 8A). In consequence of a highly significant main effect of effect of STZ treatment (*F*_(1,25)_ = 12.715, p = 0.001) and a significant interaction between STZ and LIR treatment (*F*_(1,25)_ = 10.075, p = 0.004), subsequent Bonferroni multiple comparison tests revealed that *Glut1* relative expression levels were reduced in the STZ/SAL group compared to the VEH/SAL group with high significance (p = 0.0002) and that after LIR treatment Glut1 mRNA levels were significantly increased in the STZ group (STZ/SAL vs. STZ/LIR, p = 0.048). As also shown in Table 1 of online resource 4, statistical analyses of the relative expression of *Glut3* and *Insr* in the PFC showed only clear main effects of STZ treatment (*Glut3*: *F*_(1,25)_ = 15.603, p = 0.001) (*Insr*: *F*_(1,25)_ = 5.465, p = 0.028) with reduced expression levels of both genes in the STZ group compared to the VEH control group. Statistical analyses of *Igf1* relative expression data in the PFC however showed a main effect of STZ treatment (Igf1: *F*_(1,25)_ = 6.809, p = 0.015), too, but with increased *Igf1* expression levels in the STZ group compared to the VEH control group (Fig. 8A). Although the graphs suggest that the LIR treatment has a positive/increasing effect on the expression of *Glu3* and *Insr*, statistical analysis reveals no significant differences between the STZ/SAL and STZ/LIR groups.

As also shown in Table 1 of online resource 4 statistical analyses of the relative expression data of *Glut1*, *Glut3*, *Insr* and Igf1 in the Hippocampus showed only clear main effects of STZ treatment (*Glut1*: F(1,25) = 20.354, p = < 0.0001; *Glut3*: *F*_(1,25)_ = 28.538, p < 0.0001; *Insr*: *F*_(1,25)_ = 28.784, p = < 0.0001; *Igf1*: *F*_(1,25)_ = 7.222, p = 0.013) with reduced expression levels of both genes in the STZ group compared to the VEH control group. Although the graphs with qPCR results of PFC as well as Hippocampus suggest that the LIR treatment has a positive/increasing effect on the expression of *Glu3* and *Insr* in the PFC and of *Glut1*, *Glut3*, and *Insr* in the Hippocampus statistical analysis reveals no significant differences between the STZ/SAL and STZ/LIR groups in all cases.

### LIR treatment seems to stimulate the expression of genes involved in neuroinflammation, mainly in STZ treated rats.

**Fig. 9:**
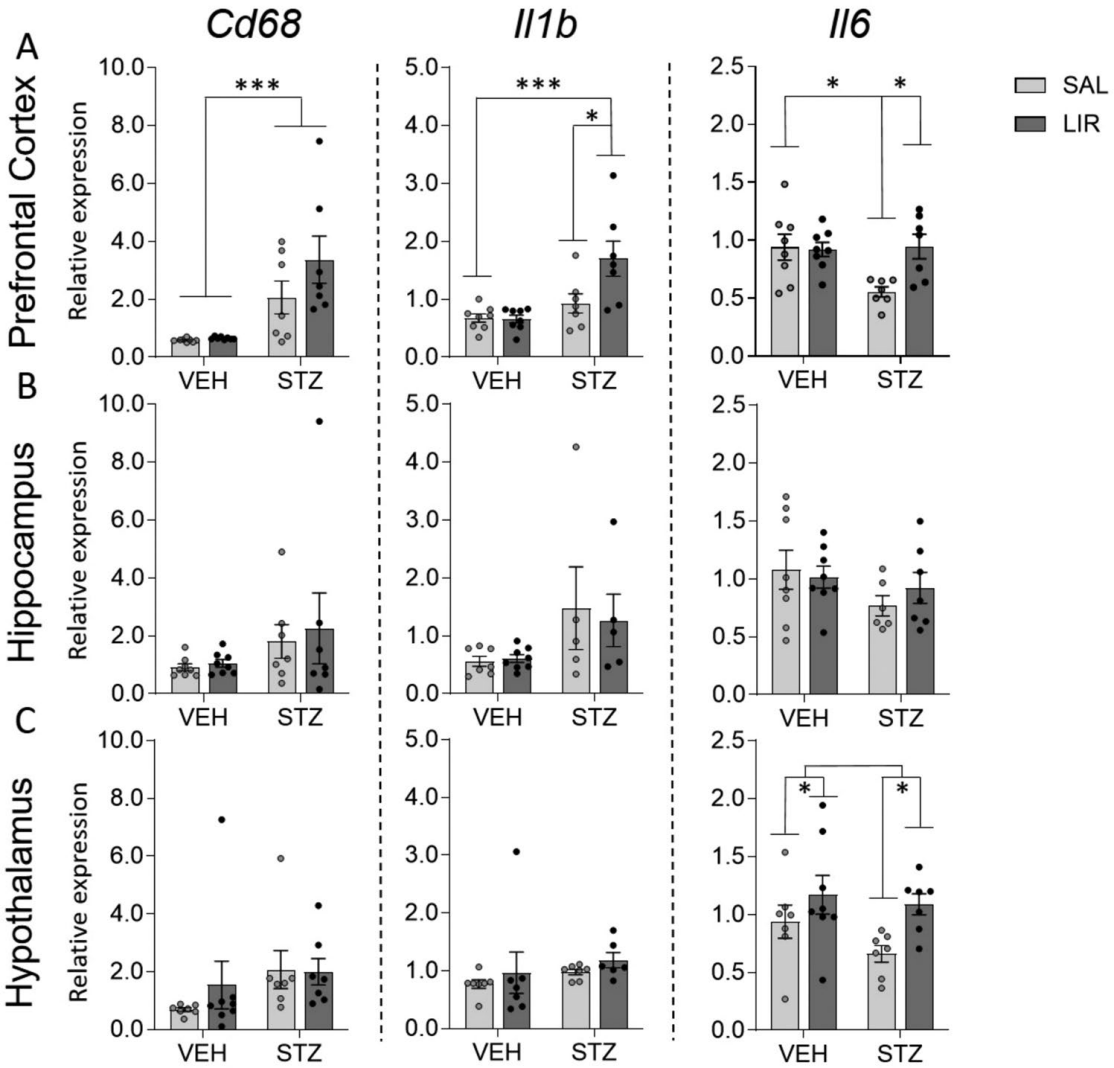
Expression levels of genes related to inflammation such as *Cd 68*, *Interleukin 1-ß (Il1b),* and *interleukin 6 (Il6)* in Prefrontal cortex (**A**), Hippocampus (**B**), and Hypothalamus (**C**). A two-way ANOVA test with Bonferroni’s multiple comparison test as a post hoc-test was applied. A significant group by treatment interaction could only be detected in the prefrontal cortex using primer pairs specific for Il1b and Il6. Data are shown as individual dots and additionally mean ± SEM with ^*^p ≤ 0.05, ^***^p < 0.001

In addition to the above-mentioned investigations of expression levels of glucose metabolism- and insulin system-related genes, qPCR with primer pairs specific for a few genes that play an important role in neuroinflammation were performed with RNA obtained from PFC, hippocampus, caudate putamen, and hypothalamus using qPCR. Results obtained from two-way mixed ANOVA with relative expression levels of inflammation-related genes such as *the cluster of Differentiation 68* (*Cd68), interleukin 1 beta* (*Il1b*), and *interleukin 6* (*Il6*) are summarized in the Table of online resource 4. Figure 9 graphically presents gene expression results of PFC, Hippocampus, and Hypothalamus. As shown in Fig. 9 most gene expression differences between groups could be detected in PFC (Fig. 9A). In PFC, results obtained from two-way mixed ANOVA on the relative expression data of *Cd68* showed a highly significant main group effect of STZ treatment (*F*_(1,25)_ = 22.69, p = < 0.0001). Statistical analysis of *Il1b* gene expression levels revealed a highly significant main group effect of STZ treatment (*F*_(1,25)_ = 15.94, p = 0.001), but also a significant interaction between groups and LIR treatment (*F*_(1,25)_ = 4.262, p = 0.05). Subsequent Bonferroni multiple comparison tests revealed that *Il1b* relative expression levels were increased in the STZ/LIR group compared to the STZ/SAL group (p = 0.0238) (a consequence of LIR treatment) and in the STZ/LIR group compared to the VEH/LIR (0 = 0.0004) (different effects of LIR in VEH- and STZ-treated animals), whereas no expression differences could be shown between both VEH groups (VEH/SAL vs. VEH/LIR). LIR treatment also showed an inflammation-promoting effect on *Il6* expression, but exclusively in STZ-icv-treated rats. Results obtained from two-way mixed ANOVA on the relative expression data of *Il6* revealed a significant interaction between groups and LIR treatment, with increased expression levels in the STZ/LIR group compared STZ/SAL (p = 0.0165) but no effects of LIR on expression levels of *Il6* in VEH-treated rats (between VEH/LIR and VEH/SAL). In addition, STZ treatment results in reduced expression levels of *Il6* in the STZ/SAL group compared to the VEH/SAL group (p = 0.0147).

In the Hippocampus (Fig. 9B) no significant differences between individual groups were detected although STZ treatment seems to have a stimulatory effect on the expression of Cd68 and Il1b, but not on Il6. On the contrary, STZ treatment appears to have an inhibitory effect on the expression of Il6.

The graphical representation of the gene expression results of the hypothalamus (Fig. 9C) also indicate a stimulatory influence of STZ on the expression of Cd68 and Il1b, whereas STZ seems to have a rather inhibitory influence on the expression of Il6. All these differences did not reach the level of significance. However, statistical analysis of Il6 expression revealed a significant main effect of LIR treatment (*F*_(1,25)_ = 6.487, p = 0.018), regardless of VEH or STZ treatment.

## Discussion

LIR is not only widely used for the treatment of T2DM but is now increasingly used to treat obesity (Robinson et al. 2013). The press often promotes incretin-based therapy as a miraculous weight loss solution for individuals with and without diabetes, a claim that merits critical examination. As expected, we were able to demonstrate weight loss through LIR treatment, an effect that has also been shown in other studies (Raun et al. 2007; Knudsen 2010; Buganova et al. 2017). According to Mashayekhi et al. (2024), a study with obese people who were at risk of developing diabetes showed that LIR treatment increased insulin sensitivity and decreased fasting and post-meal glucose levels before weight loss. In animal models such as C57BL/6 mice, LIR treatment was shown to reduce body and fat pads weight as well as blood glucose and triglyceride levels in diet-induced obesity (Buganova et al. 2017). Recently, as GLP-1 receptor agonists have become increasingly popular as weight loss drugs, it is crucial to investigate their potential long-term effects, particularly any adverse ones. In our study, in all experimental rats, plasma glucose concentrations were in the normal physiological range in the STZ-icv and control groups (3.9–5.6 mmol/L, Wang et al. 2010). This is in accordance with other studies showing that central administration of low STZ doses (1–3 mg/kg, icv) results in a normal range of blood glucose levels (Nitsch and Hoyer 1991; Plaschke and Hoyer 1993; Duelli et al. 1994) and does not produce diabetes mellitus (with plasma glucose levels ≥ 14 mmol/l, Omolaoye et al. 2018). The GLP-1 receptor agonist LIR generally shows the potential of improving insulin sensitivity and decreasing fasting and postprandial glucose concentrations (Mashayekhi et al. 2024). Moreover, LIR has been demonstrated to decrease cardiovascular morbidity and mortality in patients with type 2 diabetes and high cardiovascular risk (Marso et al. 2016). That LIR treatment had no reducing effect on these normal blood glucose levels of our experimental rats is not surprising, as it is shown that incretins do only reduce blood glucose levels in individuals with high plasma glucose concentrations, such as people with type II diabetes, and not in normoglycemic people (Vella et al. 2002). 

The aim of our STZ-icv-LIR study was to show whether the antidiabetic drug LIR has the potential to reverse behavioral and neurobiological consequences of STZ-icv treatment of rats, which is acknowledged as an animal model for the sporadic form of AD.

In line with expectations and published reports of STZ-icv-induced cognitive deficits (Lannert and Hoyer 1998; Prickaerts et al. 1999, 2000; Sharma and Gupta 2001; Hoyer 2004; Salkovic-Petrisic et al. 2006; Grunblatt et al. 2006/2007) decreased memory function was found with 3 mg STZ-icv-treated rats of this study in MWM test. Similar cognitive deficits could be shown with different doses of STZ and seem to be long-term (observed as early as 2 weeks after STZ-icv administration and are maintained up to 9 weeks post-treatment) and progressive (Mayer et al. 1990, Blokland and Jolles 1994; Lannert and Hoyer 1998; Salkovic-Petrisic et al. 2006; Grünblatt et al. 2006; Shoham et al. 2007; Knezovic et al. 2015). The GLP-1 analogue LIR has shown neuroprotective effects in a range of animal models of neurodegenerative disorders, such as AD (McClean et al. 2011; McClean and Holscher 2014; Hansen et al. 2015), Parkinson’s disease (Badawi et al. 2017; Liu et al. 2015), and status epilepticus (Wang et al. 2018). Whereas LIR significantly improved the memory ability and neurological dysfunction of Sevoflurane-treated rats, a model of postoperative cognitive function (Hu et al. 2024) and of APP/PS1 mice, a model for the familial form of AD (McClean et al. 2011; for review: Wicinski et al. 2019), we were not able to show that LIR reverses this deteriorating STZ-icv-effect, e.g. by reducing the number of errors.

Furthermore, we showed that treatment with both STZ-icv and LIR resulted in impairment of passive avoidance behavior, as step-through latency in the 24-h memory retention test of the PA test was reduced additively by both treatments, albeit only at trend level. Our findings of impaired passive avoidance behavior after STZ-icv treatment are corroborated with earlier reports using rats and mice as animal models (Mayer et al. 1990; Lannert and Hoyer 1998; Ishrat et al. 2006, 2009) or appear to be unaltered as shown in mouse STZ-icv and 5xFAD mouse models compared to controls (Blokland and Jolles 1993; Paladugu et al. 2021). That LIR treatment reduced this step-through latency even further, indicating an additive negative effect of STZ-icv and LIR on fear memory performance, was contrary to our expectations, and has not been described in other studies yet. In contrast, Palleira and coworkers demonstrated an improvement in learning and memory following LIR treatment, supported by an increased step-through latency of LIR-treated rats in the PA test (Palleria et al. 2017). However, Palleira proposed a preventive effect of LIR treatment on cognitive decline associated with STZ-icv treatment. The possible reason may lie in the time of the LIR treatment initiation after STZ-icv injection. In our previous research we have demonstrated distinct stages of STZ-icv rat model disease progression: partially reversible, acute cognitive deficit and neurochemical alterations in the early post STZ-icv period (around 1 month); partial normalization and compensation (1–3 months) and a slow but progressive chronic decline seen from 3 months after STZ-icv treatment (Knezovic et al. 2015). Furthermore, in our previous research we have demonstrated that the therapeutic effects of oral galactose on cognitive deficits seem to be dependent on the stage and/or severity of the AD-like pathology, since the cognitive improvement was previously seen in the early (treatment started 1 month after STZ-icv injection; Knezovic et al. 2018) but not in the advanced stage of sAD (treatment started 4 months after STZ-icv; Babic-Perhoc et al. 2019). On the other hand, LIR beneficial effect on cognition may be due to severity of cognitive deficit depending on the STZ dose used. Some STZ-icv dose-dependency has been suggested with lower STZ doses inducing less severe cognitive deficits (Blokland and Jolles 1994; Prickaerts et al. 2000; Grünblatt et al. 2006; Knezovic et al. 2015).

 AN in the hippocampus is a complex process involved in memory formation and is known to be altered in AD (for review: Salta et al. 2023). Here we were able to demonstrate the negative effect of STZ-icv treatment on hippocampal AN not only at the protein level by applying a quantitative immunohistochemistry study using an antibody detecting the DCX protein but also at the mRNA level by analyzing the expression levels of the gene *NeuroD1*, a bHLH transcription factor involved in neuronal differentiation, in addition to the *Dcx* gene. Results obtained at both levels confirmed each other and showed that STZ-icv treatment has a negative effect on AN. Decreased density of DCX-positive cells detected in groups treated with STZ aligns with the results of other studies obtained with the former BrdU technique (Sun et al. 2015, 2018). Additionally, it was shown that STZ treatment of NSCs caused elevated levels of ROS and impaired cell proliferation and differentiation (Qu et al. 2012). Another study showed that STZ induced an inflammatory response with microgliosis and astrogliosis in the hippocampus, which resulted in the reduction of the proliferation of NSCs in the DG and decreased survival of newborn neurons. Our findings, accompanied by the results of other studies, indicate that STZ-induced neuroinflammation plays a negative role in AN (Bassani et al. 2018; Mishra et al. 2018). Moreover, the negative correlation we revealed between the density of DCX-positive neurons and MWM performance suggests that reduced AN as well as neuroinflammation are involved in the impairment of spatial memory in this STZ-icv sAD model.

Just as we could not demonstrate a restorative effect of LIR on spatial learning impairment in STZ-icv treated, we could also not demonstrate a reversal effect of LIR on reduced AN in the hippocampus of STZ-icv treated rats. This does not meet our expectations, as it was reported in previous studies that LIR is able to promote the birth of new neurons in the hippocampus, e.g. in APPswe/PS1∆E9 mice, an animal model for the early-onset familial form of AD (McClean et al. 2011; Parthsarathy & Hölscher 2013). One reason for the lack of positive effect of LIR treatment on AN could be the longer treatment duration, e.g. in McClean’s study, which treated the mice for 56 days, rather than just 30 days as in our study (McClean et al. 2011). However, a study with another incretin analogue, Lixisenatide, given only for 3 weeks (28 days), could also show increased cell proliferation and the number of newborn neurons in the hippocampus compared to the saline control (Hunter and Hölscher 2012). LIR also promotes AN in mice with induced diabetes, as assessed by Ki67 immunostaining of the hippocampus (Zhao et al. 2022).

Neuroinflammation is a prominent and early feature of AD, which involves the activation of glial cells represented by a highly heterogeneous population of mostly microglia and astrocytes (Ferreira et al. 2014, 2018; Ransohoff 2016). IL1B is a proinflammatory cytokine, a member of the interleukin 1 cytokine family, which regulates the expression of other pro-inflammatory cytokines. IL1B levels are elevated in AD and it was shown that IL1B participates in the synthesis of β-amyloid because it acts as a regulator of APP synthesis (Twarowski and Herbet 2023). Moreover, a link between STZ icv treatment effects and another major pathophysiological protein process in AD, the abnormal hyperphosphorylation of tau proteins, has also been demonstrated. STZ is shown to increase the expression of the *caspase* 1 gene and to increase the activity of the caspase 1 protein (also known as interleukin-1 converting enzyme; ICE). Upon activation through integration into a caspase-1-containing inflammasome, this enzyme cleaves the precursors of IL-1β and IL-18 into their active neuropeptide forms, leading to increased tau hyperphosphorylation and the propagation of neuroinflammation (Li et al. 2020a). Vice versa, extracellular tau stimulates phagocytosis of living neurons by activated microglia via Toll-like 4 receptor–NLRP3 infammasome–caspase-1 signalling axis (Pampuscenko et al. 2023). As high protein expression of CD68 indicates activation of microglia in the brain, it is often also used as a marker of neuroinflammation (*CD68* is a lysosomal protein expressed in high levels by macrophages and activated *microglia* and in low levels by resting *microglia*). It was previously shown that CD68 protein levels were increased in *APOE e4* allele carriers, and it was strongly associated with dementia and poor cognitive functioning (Minett et al. 2016). IL-6 is a multifunctional cytokine with various roles outside and inside of the CNS (Erta et al. 2012). Therefore, we expected to see an increased expression of the inflammation markers *Cd68*, *Il1b* and *Il6* in groups treated with STZ and a decreased expression in groups having LIR treatment (at least in the STZ-icv groups). Increased expression of *Cd68* and *Il1b* as a consequence of STZ-icv treatment, which was most evident in the PFC and caudate putamen, aligns with our expectations and literature. However, the qPCR results using primer pairs specific for *Il6* let to unexpected results, as *Il6* expression was found to be reduced in STZ/SAL treated compared to VEH/SAL treated rats. Though IL-6 is usually regarded as a proinflammatory cytokine, at low levels it can act as an anti-inflammatory myokine as it regulates neuronal survival and acts as an immunosuppressant by inhibiting interferon-g, IL1B, and lipopolysaccharide-induced synthesis of TNF-α protein in glial cells (Scheller et al. 2011; Erta et al. 2012; Trapero & Cauli 2014). IL-6 may stimulate astrocytes to nerve growth factor (NGF) production and act by itself as neurotrophic factor in synergy with NGF (Frei et al. 1989), but also independent of the action of NGF (Hama et al. 1989). Also, IL-6 can induce neuronal differentiation (Satoh et al. 1988). It is also suggested that IL-6 is involved in the repair of the consequences of traumatic brain injuries and in cognitive dysfunction (Trapero and Cauli 2014). However, during illness or other pathological conditions, IL-6’s proinflammatory property are discussed to dominate over its beneficial functions, and it may lead to chronic neuroinflammation, which is harmful for a brain: negative effects on neurogenesis and neurodegeneration (Shan et al. 2024). But, as normal serum IL-6 levels were found in the early stages of late-onset sporadic AD (van Duijn et al. 1990;) and as IL-6 secretion is found to be decreased in severely demented patients versus controls (suggesting that it might negatively correlate with the severity of dementia; Kalman et al. 1997; Sala et al. 2003), the AD subtype and the stage of disease might explain the discrepancies observed between studies. Also interesting in this context is that patients with late-onset AD have significantly higher IL-6 levels compared to those with early-onset AD in which a rapid and more severe progression of the disease occurs (Jellinger 2009). On this basis, the reduction in Il6 expression after STZ-icv treatment can be interpreted the following: the STZ-icv-treated rats in this study represent a later, severe stage of AD. Moreover, the complex regulation of *IL6/Il6* gene expression may at least partially explain the different effects of IL-6, as it is delivered through different binding sites for transcription factors, including those for nuclear factor-κB (NF-κB), nuclear factor IL-6 (NF-IL-6), and activator protein-1, as well as two glucocorticoid-responsive elements (GRE 1 and GRE2) (Dendorfer et al. 1994).

As treatment with the GLP-1 analogue LIR, on the other hand, is proposed to have an attenuating effect on neuroinflammation, and as inflammation and brain insulin resistance affect the other in a positive feedback loop, our results of a promoting effect of LIR on *Il1b* and *Il6* expression in the STZ-icv treated groups in the PFC and of a general reinforcing effect on *Il6* expression in the hypothalamus were unexpected. LIR has been shown to be able to pass the blood–brain barrier (Hunter and Hölscher 2012) and possesses neuroprotective properties, such as a reducing effect on microglia and/or astrocyte activation as well as on amyloid ß and hyperphosphorylated tau (McClean et al. 2011; Xiong et al. 2013; Paladugu et al. 2021). Paladugu and coworkers propose that LIR is able to reduce early neuropathological markers before obvious memory deficits occur (Paladugu et al. 2021). In more detail, LIR reduced by 50% the number of activated microglia in the cortex and the Hippocampus of APP/PS1 mice (McClean et al. 2011); it also prevents astrocytosis in APPswe/PS1dE9 mice (Diz-Chaves et al. 2022). Another GLP-1 receptor agonist, exendin-4 also reduced the mRNA expression of *Il1b* and Tnf-α, but not Il6 in lipopolysaccharide induced microglia inflammation (Lee et al. 2018b, a). The anti-inflammatory effect of LIR works through cAMP regulation of MAPK and JNK pathways (Duffy and Hölscher 2013; Xiong et al. 2013; McClean et al. 2015) and by modulating insulin receptors (Long-Smith et al. 2013; Batista et al. 2018). The increased expression of *Il6* in groups treated with LIR could be explained at least to a certain extent by its dual role/function as pro- as well as anti-inflammatory. As suggested by Scheller and coworkers regenerative or anti-inflammatory activities of IL-6 are mediated by classic signaling whereas pro-inflammatory responses of interleukin-6 are rather mediated by trans-signaling (Scheller et al. 2011). IL-6 and soluble IL-6 receptor (sIL-6R) activation of glycoprotein (gp)130 represent the trans-signaling pathway and it is shown that IL-1B is able to increase the levels of sIL-6R (Franchimont et al. 2005). Gutierrez et al. discover that GLP1 analogs induce IL-6 secretion by monocytes and show that IL-6 signaling in adipocytes activates brown adipogenesis and thermogenesis that contributes to anti-diabetic effects of GLP1 analogs (Gutierrez et al. 2022). In humans, GLP1 signaling elevates circulating IL-6 activating STAT3 in preadipocytes. As a fulcrum of many vital cellular processes, the JAK/STAT pathway constitutes a rapid membrane-to-nucleus signaling module and induces the expression of various critical mediators of cancer and inflammation (Hu et al. 2021). Therefore, we hypothesize that LIR treatment may lead to increased *Il6* expression levels in the LIR-treated groups, as LIR can activate IL-6 proinflammatory signaling pathways involving components of the trans signaling pathway and activation of the JAK/STAT pathway.

Nevertheless, IL-6 and its expression following STZ and LIR treatment should be further investigated.

The strongest effects on the expression of glucose metabolism- and insulin system-related genes, such as *Glut1/3*, *Insr*, *Igf1*, *Irs1* were the result of STZ-icv treatment and were almost exclusively detected in the PFC and hippocampus. In both brain regions, STZ-icv treatment resulted in lower expression levels of *Glut1*, *Glut3,* and *Insr* compared to the controls, but regarding *Igf1*, a decreased level of expression was only observed in the hippocampus, while it was even increased in the PFC of STZ-icv-treated rats. We also analyzed the expression levels of *Glut4*, an insulin-dependent glucose transporter that in the brain plays a role in rapidly providing additional glucose to neurons under conditions of high-energy demand (Apelt et al. 1999; McEwen and Reagan 2004; Grillo et al. 2009). Our result that *Glut4* expression is not influenced by STZ-icv treatment is in accordance with unaltered GLUT4 protein levels in human AD brains (Liu et al. 2008; and correlates to abnormal hyperphosphorylation of tau) and in NSE/hPS2m Tg mice, a transgenic mouse model of AD (Lee et al. 2013), but is in contrast to the findings of Chen and coworkers, who demonstrated reduced GLUT4 protein concentrations in STZ-icv treated male Sprague–Dawley rats compared to controls (Chen et al. 2021). Furthermore, it should be noted that activation of glucose uptake by insulin stimulation occurs primarily via the translocation of GLUT4 protein to the plasma membrane. In the hippocampus the functional anatomical substrate of insulin signaling is shown to be GLUT4 colocalized with the INSR in neurons. And as already shown in peripheral tissues (such as adipose cells and skeletal muscle cells) in hippocampal neurons, activation of intracellular signaling events also leads to the translocation of GLUT4 to the plasma membrane, resulting in increased glucose uptake into hippocampal neurons (Grillo et al. 2009; Brewer et al. 2014). Therefore, gene expression levels or the total amount of cellular protein (including intracellular pools) may not reflect functional changes in glucose transport. As not only insulin, but also LIR results in the translocation of GLUT4 to the plasma membrane (Li et al. 2014) the lack of significant changes in *Glut4* gene expression in this study as a result of both STZ-icv treatment and LIR treatment is not unexpected.

The same reducing effect in consequence of STZ-icv treatment was found with analyzing the expression of genes coding for downstream proteins of the insulin/IGF-signaling pathway, such as *Gsk3b*, *Pik3ca*, *Rps6kb1*, and *Mtor*.

These findings align with the established role of impaired brain insulin signaling and abnormal cerebral glucose metabolism in sAD pathology (Liu et al. 2008; Correia et al. 2011; Kyrtata et al. 2021). Our qPCR results are essentially consistent with relative mRNA levels reported in other studies using STZ-icv treated rats or monkeys (Lee et al. 2013, Lee et al. 2014, Grünblatt et al. 2004, Lester-Coll et al. 2006). At the protein level, the observed reductions of GLUT1 and GLUT 3 levels in AD animal models compared to controls, both in STZ-icv treated rats (Deng et al. 2009; Salkovic-Petrisic et al. 2014) and NSE/hPS2m Tg mice (Lee et al. 2013), mirror the mRNA-level differences. However, in most studies, protein concentrations of INSR and IRS1 were shown to be similar in STZ-icv treated animals and controls (Deng et al. 2009; Knezovic et al. 2017; dos Santos et al. 2018; Mishra et al. 2018), which contrasts with our qPCR results. This discrepancy may be due to various post-transcriptional modifications that occur during the translation process.

LIR was only able to significantly reverse the negative effect of STZ-icv treatment on the expression of *Glut1* in PFC. This enhancing and restorative effect of LIR on *Glut1* expression is in line with the literature data. Gejl and colleagues demonstrated via FDG-PET-MRI that treatment with GLP-1 analogues restores glucose transport at the blood–brain barrier, which is typically impaired in AD patients (Gejl et al. 2017). This effect is not limited to the brain, in skeletal muscle cells, both the GLP-1 receptor agonists exenatide and LIR have been shown to activate glucose transport at L6 skeletal muscle myotubes via an AMPK-dependent mechanism (Andreozzi et al. 2016). The insulin-sensitive GLUT4 could have a role in this effect, as LIR seems to translocate the GLUT4 protein to the plasma membrane of mouse skeletal muscle cells (Li et al. 2014). Overall, the limited effect of LIR on *Glut1*—and not on other glucose metabolism or insulin signaling-related genes—is not entirely unexpected. This is consistent with our behavioral data from the MWM test, which showed no clear improvement in learning and memory following LIR treatment.

## Conclusion

In this study using the STZ-icv rat model, we clearly demonstrated increased neuroinflammation and disruptions in the expression of genes involved in brain glucose metabolism and the insulin/IGF1 receptor signaling cascade, both considered key features of the pathophysiology of sAD. Restoring the function of these metabolism- and insulin signaling-related pathways, e.g. by treatment with antidiabetic drugs such as the incretin LIR, may represent a promising strategy to prevent disease progression in AD. Although we did not observe a significant effect of LIR on STZ-icv–induced cognitive impairment in this study, we did find that LIR partially restored dysregulated gene expression, particularly in genes related to glucose metabolism. Future studies employing a more refined experimental design, e.g., different dosing of STZ and LIR, will help to further clarify the therapeutic potential of LIR in sAD. 

## Supplementary Information

Below is the link to the electronic supplementary material.

Supplementary file1 (DOCX 16 KB)

Supplementary file2 (DOCX 13 KB)

Supplementary file3 (DOCX 19 KB)

Supplementary file4 (DOCX 41 KB)

## Data Availability

Not applicable.
