# Supplementary material for: Approaching therapy of Alzheimer’s disease via the antidiabetic drug liraglutide—a study with streptozotocin intracerebroventricularly treated Wistar rats
Source: J Neural Transm (Vienna). 2025 Jul 12;132(10):1587–608. doi: 10.1007/s00702-025-02979-z (PMC12568868; doi:10.1007/s00702-025-02979-z)
Supplement: Supplementary file 1 — Supplementary file1 (DOCX 16 KB) [file 702_2025_2979_MOESM1_ESM.docx]

**Online Resource 1**

Table 1: **List of self-designed primer pairs of target or reference genes for qPCR.** Presented are the gene name with the corresponding gene symbol as well as the amplicon size (bps), information about the forward (F) and reverse (R) sequence of the oligonucleotides and the GeneBank accession number.

| **Gene name and symbol** | **Accession number** | **Type** | **Ampli-con size in bps** | **Primer sequence**  **(5‘ 🡪 3‘)** |
| --- | --- | --- | --- | --- |
| solute carrier family 2 member 1 (*Slc2a1*, *Glut1)* | NM_138827 | Target | 129 | (F) CTTATGTTGGCCGTGGGAGG  (R) CTCTCCATAGCGGTGGTTCC |
| solute carrier family 2 member 3 (*Slc2a3*, *Glut3*) | NM_017102 | Target | 147 | (F) GCCCCTTCTCATCTCCGTTG  (R) ATAGTGTTGACCACACCCGC |
| solute carrier family 2 member 4 (*Slc2a4*, *Glut4*) | NM_012751 | Target | 83 | (F) ACAAGATGCCGTCGGGTTT  (R) AAGGACCAGTGTCCCAGTCA |
| insulin receptor (*Insr*) | NM_017071 | Target | 115 | (F) AACAACAAGTGCATCCCCGA  (R) TCTCGCCTTCGAGGATTTGG |
| insulin receptor substrate 1 (*Irs1*) | NM_012969 | Target | 136 | (F) ACCAGAGGACCGTCAATAGC  (R) GACGTGAGGTCCTGGTTGTG |
| insulin-like growth factor 1 (*Igf1*) | NM_001082478  NM_001082479  NM_001082477  NM_178866 | Target | 104 | (F) GACGCTCTTCAGTTCGTGTG  (R) TCATCCACAATGCCCGTCTG |
| glycogen synthase kinase 3 beta (*Gsk3 b*) | NM_032080 | Target | 123 | (F) GGACAGTGGTGTGGATCAGT  (R) GGATGTGCCTTGATTTGGGG |
| phosphatidylinositol-4,5-bisphosphate 3-kinase, catalytic subunit alpha (*Pik3ca*) | NM_133399 | Target | 91 | (F) AAGGACAAGAACAAGGGCGA  (R) CCAAGATAAAGGTTGCCACGC |
| ribosomal protein S6 kinase B1 (*Rps6kb1*; *p70 S6K-alpha*) | NM_031985 | Target | 149 | (F) GGGTACTTGGTAAAGGGGGC  (R) CCGCTCTGCTTTTGTATGAGC |
| mechanistic target of rapamycin kinase (*Mtor*) | NM_019906 | Target | 131 | (F) ACGCCATGAAACACTTCGGA  (R) CAGCATCAGCTCTGGGTCAT |
| *Cd68* | NM_001031638 | Target | 74 | (F) CTTACCTTTGGATTCAAACAGGACC (R) CTGCTTGTGGGAAGGACACAT |
| Interleukin 1 β (*Il1b)* | NM_031512 | Target | 120 | (F) ACAAAAATGCCTCGTGCTGTC  (R) TCGTTGCTTGTCTCTCCTTGT |
| Interleukin 6 (*Il6)* | NM_012589 | Target | 109 | (F) AAGCCAGAGTCATTCAGAGCA  (R) AGGAGAGCATTGGAAGTTGGG |
| Cyclophilin A (*Ppia,Cyc A*) | NM_017101 | Reference | 126 | (F) TATCTGCACTGCCAAGACTGAGTG  (R) CTTCTTGCTGGTCTTGCCATTCC |
| Hypoxanthine phosphoribosyl-transferase 1 (*Hprt1*) | NM_012583 | Reference | 81 | (F) GCAGACTTTGCTTTCCTTGG  (R) CGAGAGGTCCTTTTCACCAG |
| Ribosomal protein L13A (*Rpl13A*) | NM_173340 | Reference | 132 | (F) GGATCCCTCCACCCTATGACA  (R) CTGGTACTTCCACCCGACCTC |
| Phospho-glycerate- kinase 1 (*Pgk1*) | NM_053291 | Reference | 104 | (F)ATGCAAAGACTGGCCAAGCTAC  (R)AGCCACAGCCTCAGCATATTTC |
| Tyrosine 3-monooxygenase/tryptophan 5-monooxygenase activation protein, zeta (*Ywhaz*) | NM_013011.4 | Reference | 136 | (F) TTGAGCAGAAGACGGAAGGT  (R) GAAGCATTGGGGATCAAGAA |

Table 2: **List of QuantiTect Primer assays from QIAGEN**

| **Gene name and symbol** | **Catalogue number** | **Type** | **Amplicon size in bps** | **Primer sequence**  **(5‘ 🡪 3‘)** |
| --- | --- | --- | --- | --- |
| Doublecortin (*Dcx*) | QT00196748 | Target | 129 | Not specified |
| Neuronal differentiation 1 (*NeuroD1*) | QT00408835 | Target | 147 | Not specified |
