# Supplementary material for: Approaching therapy of Alzheimer’s disease via the antidiabetic drug liraglutide—a study with streptozotocin intracerebroventricularly treated Wistar rats
Source: J Neural Transm (Vienna). 2025 Jul 12;132(10):1587–608. doi: 10.1007/s00702-025-02979-z (PMC12568868; doi:10.1007/s00702-025-02979-z)
Supplement: Supplementary file 2 — Supplementary file2 (DOCX 13 KB) [file 702_2025_2979_MOESM2_ESM.docx]

**Online resource 2**

Descriptive and explorative statistics of step-through latencies (mean±SEM) in the habituation and conditioning trials of the passive avoidance task.

|  | Mean±SEM step-through latency (s) | | | |  | Two-way ANOVA | | | | | |
| --- | --- | --- | --- | --- | --- | --- | --- | --- | --- | --- | --- |
|  | VEH/SAL | VEH/LIR | STZ/SAL | STZ/LIR |  | Group | | Treatment | | Interaction | |
|  | *n*=8 | *n*=8 | *n*=6 | *n*=7 |  | *F*(1,25) | *p* | *F*(1,25) | *p* | *F*(1,25) | *p* |
| Habituation | 44.0±5.33 | 40.0±9.77 | 29.7±4.84 | 27.7±3.43 |  | 3.894 | 0.060 | 0.195 | 0.663 | 0.023 | 0.881 |
| Conditioning | 20.4±3.46 | 18.3±3.96 | 36.8±16.4 | 19.1±2.93 |  | 1.327 | 0.260 | 1.749 | 0.198 | 1.094 | 0.306 |
