# Supplementary material for: Approaching therapy of Alzheimer’s disease via the antidiabetic drug liraglutide—a study with streptozotocin intracerebroventricularly treated Wistar rats
Source: J Neural Transm (Vienna). 2025 Jul 12;132(10):1587–608. doi: 10.1007/s00702-025-02979-z (PMC12568868; doi:10.1007/s00702-025-02979-z)
Supplement: Supplementary file 3 — Supplementary file3 (DOCX 19 KB) [file 702_2025_2979_MOESM3_ESM.docx]

**Online resource 3**

Table 1 & 2. Overview of two-way ANOVA results on different markers for adult hippocampal neurogenesis at the protein level (1) and at gene expression/mRNA level (2). Group = STZ icv vs. vehicle (VEH), Treatment = LIR vs. Saline (SAL). DCX, double cortin; MCM2, minichromosome maintenance complex component 2; NeuroD1, Neurogenic differentiation 1. Data are shown as mean±SEM.

|  |  | **Mean±SEM density of immuno-positive cells (n)** | | | | |  | **Two-way ANOVA** | |  |  |  |  |
| --- | --- | --- | --- | --- | --- | --- | --- | --- | --- | --- | --- | --- | --- |
| 1) | Protein |  | VEH/SAL | VEH/LIR | STZ/SAL | STZ/LIR |  | Group |  | Treatment |  | Interaction |  |
|  |  |  |  |  |  |  |  | F(1,25) | p | F(1,25) | p | F(1,25) | p |
|  | DCX (SGZ_anterior) | x 10-5 | 3.52 ± 0.57 (8) | 4.21 ± 0.59 (8) | 0.13 ± 0.08 (4) | 0.6 ± 0.27 (7) |  | 42.437 | **0.000** | 1.165 | 0.292 | 0.000 | 1.0 |
|  | DCX (SGZ_posterior) | x 10-5 | 4.74 ± 1.01 (8) | 5.45 ± 0.7 (7) | 0,37 ± 0.23 (4) | 1.1 ± 0.31 (7) |  | 30.724 | **0.000** | 0.839 | 0.370 | 0.000 | 1.0 |
|  | DCX (SGZ+GCL)_anterior | x 10-5 | 4.2 ± 0.58 (8) | 4.7 ± 0.58 (8) | 0.16 ± 0.06 (4) | 0.65 ± 0.31 (7) |  | 56.465 | **0.000** | 0.888 | 0.356 | 0.000 | 1.0 |
|  | DCX (SGZ+GCL)_posterior | x 10-5 | 5.65 ± 1.16 (8) | 6.61 ± 0,89 (7) | 0.63 ± 0.31 (4) | 1.28 ± 0.32 (7) |  | 31.234 | **0.000** | 0,756 | 0.394 | 0.000 | 1.0 |
|  | MCM2 (SGZ_anterior) | x 10-6 | 1.0 ± 0.00 (8) | 0.9 ± 0.00 (8) | 0,7 ± 0.00 (3) | 1.0 ± 0.00 (7) |  | 0.00 | 1.0 | 0.00 | 1.0 | 0.00 | 1.0 |
|  | MCM2 (SGZ_posterior) | x 10-6 | 1.1 ± 0.00 (8) | 0.8 ± 0.00 (8) | 0.5 ± 0.00 (4) | 0.3 ± 0.00 (6) |  | 0.00 | 1.0 | 0.00 | 1.0 | 0.00 | 1.0 |
|  | MCM2 (SGZ+GCL)_anterior | x 10-6 | 2.8 ± 0.00 (8) | 2.4 ± 0.00 (8) | 3.3 ± 0.00 (3) | 2.3 ± 0.00 (7) |  | 0.00 | 1.0 | 0.00 | 1.0 | 0.00 | 1.0 |
|  | MCM2 (SGZ+GCL)_posterior | x 10-6 | 2.6 ± 0.00 (8) | 1.9 ± 0.00 (8) | 1.5 ± 0.00 (4) | 1.1 ± 0.00 (6) |  | 0.00 | 1.0 | 0.00 | 1.0 | 0.00 | 1.0 |
|  |  |  |  |  |  |  |  |  |  |  |  |  |  |
|  |  |  | **Mean±SEM relative expression (n)** | | |  |  | **Two-way ANOVA** | |  |  |  |  |
| 2) | Genes |  | VEH/SAL | VEH/LIR | STZ/SAL | STZ/LIR |  | Group |  | Treatment |  | Interaction |  |
|  |  |  |  |  |  |  |  | F(1.25) | p | F(1.25) | p | F(1.25) | p |
|  | *Dcx* |  | 1.34 ± 0.12 (8) | 1.36 ± 0.09 (8) | 0.56 ± 0.19 (6) | 0.91 ± 0.20 (7) |  | 16.496 | **0.0004** | 1.538 | 0.226 | 1.165 | 0.291 |
|  | *NeuroD1* |  | 1.97 ± 0.16 (8) | 1.75 ± 0.26 (8) | 0.68 ± 0.20 (6) | 0.96 ± 0.29 (7) |  | 19.496 | **0.00017** | 0.015 | 0.903 | 1.134 | 0.297 |
|  |  |  |  |  |  |  |  |  |  |  |  |  |  |
