# Supplementary material for: Approaching therapy of Alzheimer’s disease via the antidiabetic drug liraglutide—a study with streptozotocin intracerebroventricularly treated Wistar rats
Source: J Neural Transm (Vienna). 2025 Jul 12;132(10):1587–608. doi: 10.1007/s00702-025-02979-z (PMC12568868; doi:10.1007/s00702-025-02979-z)
Supplement: Supplementary file 4 — Supplementary file4 (DOCX 41 KB) [file 702_2025_2979_MOESM4_ESM.docx]

**Online Resource 4**

Descriptive and explorative statistics of quantitative real-time PCR data. Shown are the data for prefrontal cortex, hippocampus, hypothalamus and caudate putamen. Statistical analysis was performed using Two-way ANOVA and as a post hoc-test the Bonferroni’s multiple comparison test.

| **Prefrontal Cortex** | | |  |  |  |  |  |  |  |  |  |  |  |
| --- | --- | --- | --- | --- | --- | --- | --- | --- | --- | --- | --- | --- | --- |
|  | **Mean±SEM relative expresssion (n)** | | | |  | **Two-way ANOVA** | |  |  |  |  |  |  |
| Genes | VEH/SAL | VEH/LIR | STZ/SAL | STZ/LIR |  | Group |  | Treatment |  | Interaction |  | Bonferroni’s multiple | |
|  |  |  |  |  |  | F(1.25) | p | F(1.25) | p | F(1.25) | p | comparison test |  |
| *Glut1* | 1.17 ± 0.06 (8) | 0.99 ± 0.13 (8) | 0.73 ± 0.19 (6) | 0.96 ± 0.19 (7) |  | 12.715 | **0.001** | 0.161 | 0.692 | 10.075 | **0.004** | **VEH/SAL vs. STZ/SAL p= 0.0002** | **STZ/SAL vs. STZ/LIR p=0.048** |
| *Glut3* | 1.23 ± 0.06 (8) | 1.08 ± 0.19 (8) | 0.70 ± 0.41 (6) | 0.86 ± 0.23 (7) |  | 15.603 | **0.001** | 0.001 | 0.981 | 2.507 | 0.126 |  |  |
| *Glut4* | 0.93 ± 0.07 (8) | 0.93 ± 0.22 (8) | 1.01 ± 0.59 (6) | 1.05 ± 0.34 (7) |  | 0.608 | 0.443 | 0.023 | 0.881 | 0.014 | 0.905 |  |  |
| *Insr* | 1.16 ± 0.04 (8) | 1.03 ± 0.26 (8) | 0.78 ± 0.37 (6) | 0.96 ± 0.28 (7) |  | 5.465 | **0.028** | 0.06 | 0.808 | 2.454 | 0.13 |  |  |
| *Irs1* | 1.15 ± 0.04 (8) | 1.01 ± 0.14 (8) | 0.79 ± 0.28 (6) | 0.98 ± 0.30 (7) |  | 5.81 | **0.024** | 0.08 | 0.78 | 4.237 | 0.05 |  |  |
| *Igf1* | 1.01 ± 0.05 (8) | 0.88 ± 0.15 (8) | 1.09 ± 0.27 (6) | 1.22 ± 0.29 (7) |  | 6.809 | **0.015** | 0 | 0.996 | 2.733 | 0.111 |  |  |
| *Gsk3*b | 1.16 ± 0.04 (8) | 0.97 ± 0.13 (8) | 0.79 ± 0.33 (6) | 0.94 ± 0.23 (7) |  | 6.626 | **0.016** | 0.086 | 0.772 | 4.85 | **0.037** | **VEH/SAL vs. STZ/SAL p=0.0057** |  |
| *Pik3*ca | 1.21 ± 0.04 (8) | 1.07 ± 0.15 (8) | 0.77 ± 0.36 (6) | 0.94 ± 0.23 (7) |  | 11.853 | **0.002** | 0.031 | 0.862 | 3.457 | 0.075 |  |  |
| *Rps6kb1* | 1.05 ± 0.03 (8) | 0.98 ± 0.10 (8) | 0.81 ± 0.31 (6) | 0.96 ± 0.17 (7) |  | 3.817 | 0.062 | 0.357 | 0.556 | 2.966 | 0.097 |  |  |
| *Mtor* | 1.15 ± 0.03 (8) | 1.02 ± 0.16 (8) | 0.78 ± 0.47 (6) | 0.99 ± 0.29 (7) |  | 4.018 | 0.056 | 0.115 | 0.738 | 2.817 | 0.106 |  |  |
| *Cd68* | 0.59 ± 0.02 (8) | 0.66 ± 0.02 (8) | 2.31 ± 0.60 (6) | 3.36 ± 0.81 (7) |  | 22.686 | **< 0.0001** | 1.455 | 0.239 | 1.118 | 0.301 |  |  |
| *Il1b* | 0.67 ± 0.07 (8) | 0.66 ± 0.07 (8) | 1.01 ± 0.17 (6) | 1.70 ± 0.30 (7) |  | 15.94 | **0.001** | 3.887 | 0.06 | 4.262 | **0.05** | **STZ/SAL vs. STZ/LIR p=0.0238** | **VEH/LIR vs. STZ/LIR p=0.0004** |
| *Il6* | 0.94 ± 0.11 (8) | 0.92 ± 0.06 (8) | 0.57 ± 0.05 (6) | 0.95 ± 0.10 (7) |  | 3.874 | 0.06 | 4.096 | 0.054 | 5.036 | **0.034** | **STZ/SAL vs. STZ/LIR p=0.0165** | **VEH/SAL vs. STZ/SAL p=0.0147** |

| **Hippocampus** | |  | |  | |  | |  | |  |  | |  | |  | |  | |  | |  |  |
| --- | --- | --- | --- | --- | --- | --- | --- | --- | --- | --- | --- | --- | --- | --- | --- | --- | --- | --- | --- | --- | --- | --- |
|  | **Mean±SEM relative expresssion (n)** | | | | | | |  | | **Two-way ANOVA** | | |  | |  | |  | |  | |  |  |
| Genes | VEH/SAL | VEH/LIR | | STZ/SAL | | STZ/LIR | |  | | Group |  | | Treatment | |  | | Interaction | |  | | Bonferroni’s multiple | |
|  |  |  | |  | |  | |  | | F(1.25) | p | | F(1.25) | | p | | F(1.25) | | p | | comparison test |  |
| *Glut1* | 1.34 ± 0.09 (8) | 1.41 ± 0.08 (8) | | 0.57 ± 0.19 (6) | | 0.95 ± 0.18 (7) | |  | | 20.354 | **<0.0001** | | 2.707 | | 0.112 | | 1.342 | | 0.258 | |  |  |
| *Glut3* | 1.51 ± 0.19 (8) | 1.41 ± 0.08 (8) | | 0.42 ± 0.18 (5) | | 0.66 ± 0.20 (7) | |  | | 28.538 | **< 0.0001** | | 0.146 | | 0.706 | | 0.964 | | 0.336 | |  |  |
| *Glut4* | 0.85 ± 0.09 (8) | 1.13 ± 0.11 (8) | | 0.70 ± 0.23 (4) | | 0.81 ± 0.14 (6) | |  | | 2.842 | 0.106 | | 0.08 | | 0.168 | | 0.366 | | 0.551 | |  |  |
| *Insr* | 1.31 ± 0.13 (8) | 1.43 ± 0.05 (8) | | 0.43 ± 0.15 (5) | | 0.72 ± 0.22 (7) | |  | | 28.784 | **<0.0001** | | 1.954 | | 0.175 | | 0.296 | | 0.591 | |  |  |
| *Irs1* | 1.37 ± 0.09 (8) | 1.28 ± 0.04 (8) | | 0.53 ± 0.20 (5) | | 0.78 ± 0.17 (7) | |  | | 26.905 | **<0.0001** | | 0.357 | | 0.556 | | 1.573 | | 0.222 | |  |  |
| *Igf1* | 1.16 ± 0.04 (8) | 1.07 ± 0.04 (8) | | 0.87 ± 0.20 (6) | | 0.78 ± 0.14 (6) | |  | | 7.222 | **0.013** | | 0.654 | | 0.427 | | 0.002 | | 0.966 | |  |  |
| *Gsk3*b | 1.38 ± 0.12 (8) | 1.52 ± 0.11 (8) | | 0.44 ± 0.15 (6) | | 0.86 ± 0.21 (7) | |  | | 27.503 | **<0.0001** | | 3.348 | | 0.079 | | 0.827 | | 0.372 | |  |  |
| *Pik3*ca | 1.51 ± 0.13 (8) | 1.46 ± 0.06 (8) | | 0.42 ± 0.14 (6) | | 0.81 ± 0.20 (7) | |  | | 40.96 | **< 0.0001** | | 1.574 | | 0.221 | | 2.667 | | 0.115 | |  |  |
| *Rps6kb1* | 1.28 ± 0.06 (8) | 1.35 ± 0.05 (8) | | 0.57 ± 0.15 (6) | | 0.89 ± 0.16 (7) | |  | | 30.079 | **<0.0001** | | 3.302 | | 0.081 | | 1.397 | | 0.248 | |  |  |
| *Mtor* | 1.63 ± 0.18 (8) | 1.60 ± 0.09 (8) | | 0.37 ± 0.15 (6) | | 0.81 ± 0.26 (7) | |  | | 33.282 | **< 0.0001** | | 1.342 | | 0.258 | | 1.777 | | 0.195 | |  |  |
| *Cd68* | 0.91 ± 0.12 (8) | 1.05 ± 0.13 (8) | | 1.94 ± 0.67 (6) | | 1.07 ± 0.81 (6) | |  | | 2.426 | 0.132 | | 1.195 | | 0.285 | | 2.248 | | 0.147 | |  |  |
| *Il1b* | 0.56 ± 0.09 (7) | 0.60 ± 0.07 (8) | | 1.62 ± 0.90 (4) | | 0.84 ± 0.39 (4) | |  | | 3.836 | 0.065 | | 1.233 | | 0.281 | | 0.002 | | 0.225 | |  |  |
| *Il6* | 1.08 ± 0.17 (8) | 1.01 ± 0.10 (8) | | 0.77 ± 0.11 (5) | | 0.92 ± 0.13 (7) | |  | | 2.093 | 0.161 | | 0.098 | | 0.757 | | 0.606 | | 0.444 | |  |  |
| **Hypothalamus** | |  |  | |  | |  | |  | | |  | |  | |  | |  | |  |  |  |
|  | **Mean±SEM relative expresssion (n)** | | | | | |  | | **Two-way ANOVA** | | | | |  | |  | |  | |  |  |  |
| Genes | VEH/SAL | VEH/LIR | STZ/SAL | | STZ/LIR | |  | | Group | | |  | | Treatment | |  | | Interaction | |  | Bonferroni’s multiple | |
|  |  |  |  | |  | |  | | F(1.25) | | | p | | F(1.25) | | p | | F(1.25) | | p | comparison test |  |
| *Glut1* | 1.17 ± 0.11 (7) | 1.17 ± 0.17 (8) | 0.95 ± 0.12 (6) | | 1.05 ± 0.14 (7) | |  | | 1.426 | | | 0.244 | | 0.144 | | 0.708 | | 0.118 | | 0.734 |  |  |
| *Glut3* | 1.16 ± 0.17 (7) | 1.12 ± 0.23 (8) | 1.35 ± 0.32 (6) | | 0.94 ± 0.21 (7) | |  | | 0.000057 | | | 0.994 | | 0.87 | | 0.36 | | 0.611 | | 0.442 |  |  |
| *Glut4* | 0.99 ± 0.07 (7) | 1.04 ± 0.19 (8) | 1.25 ± 0.31 (6) | | 1.09 ± 0.16 (7) | |  | | 0.656 | | | 0.426 | | 0.08 | | 0.78 | | 0.29 | | 0.595 |  |  |
| *Insr* | 1.19 ± 0.17 (7) | 1.35 ± 0.30 (8) | 1.07 ± 0.18 (6) | | 1.03 ± 0.20 (7) | |  | | 0.896 | | | 0.353 | | 0.08 | | 0.78 | | 0.181 | | 0.674 |  |  |
| *Irs1* | 1.30 ± 0.13 (7) | 1.30 ± 0.21 (8) | 1.04 ± 0.11 (6) | | 1.12 ± 0.16 (7) | |  | | 1.687 | | | 0.206 | | 0.052 | | 0.822 | | 0.062 | | 0.805 |  |  |
| *Igf1* | 0.97 ± 0.07 (7) | 1.15 ± 0.10 (8) | 1.28 ± 0.15 (6) | | 1.19 ± 0.11(7) | |  | | 2.737 | | | 0.111 | | 0.186 | | 0.67 | | 1.561 | | 0.224 |  |  |
| *Gsk3*b | 1.19 ± 0.12 (7) | 1.22 ± 0.21 (8) | 1.06 ± 0.16 (6) | | 1.02 ± 0.16 (7) | |  | | 0.943 | | | 0.341 | | 0.00035 | | 0.985 | | 0.036 | | 0.851 |  |  |
| *Pik3*ca | 1.19 ± 0.16 (7) | 1.22 ± 0.22 (8) | 1.12 ± 0.21 (6) | | 1.02 ± 0.18 (7) | |  | | 0.481 | | | 0.495 | | 0.04 | | 0.844 | | 0.11 | | 0.743 |  |  |
| *Rps6kb1* | 0.64 ± 0.05 (7) | 0.62 ± 0.06 (8) | 1.39 ± 0.15 (6) | | 1.41 ± 0.18 (7) | |  | | 44.189 | | | **< 0.0001** | | 0.00044 | | 0.983 | | 0.027 | | 0.871 |  |  |
| *Mtor* | 1.12 ± 0.17 (7) | 1.23 ± 0.29 (8) | 1.16 ± 0.21 (6) | | 0.99 ± 0.23 (7) | |  | | 0.204 | | | 0.655 | | 0.015 | | 0.904 | | 0.35 | | 0.56 |  |  |
| *Cd68* | 0.69 ± 0.07 (7) | 1.54 ± 0.83 (7) | 2.28 ± 073 (6) | | 1.99 ± 0.46 (7) | |  | | 2.770 | | | 0.109 | | 0.204 | | 0.656 | | 0.849 | | 0.366 |  |  |
| *Il1b* | 0.77 ± 0.08 (7) | 0.96 ± 0.36 (7) | 1.01 ± 0.04 (6) | | 1.19 ± 0.13 (6) | |  | | 1.228 | | | 0.28 | | 0.798 | | 0.381 | | 0.002 | | 0.966 |  |  |
| *Il6* | 0.94 ± 0.14 (7) | 1.17 ± 0.17 (8) | 0.64 ± 0.08 (6) | | 1.09 ± 0.09 (7) | |  | | 2.005 | | | 0.17 | | 6.487 | | **0.018** | | 0.643 | | 0.431 |  |  |

| **Caudate putamen** | | |  |  |  |  |  |  |  |  |  |  |  |
| --- | --- | --- | --- | --- | --- | --- | --- | --- | --- | --- | --- | --- | --- |
|  | **Mean±SEM relative expresssion (n)** | | | |  | **Two-way ANOVA** | |  |  |  |  |  |  |
| Genes | VEH/SAL | VEH/LIR | STZ/SAL | STZ/LIR |  | Group |  | Treatment |  | Interaction |  | Bonferroni’s multiple | |
|  |  |  |  |  |  | F(1.25) | p | F(1.25) | p | F(1.25) | p | comparison test |  |
| *Glut1* | 0.92 ± 0.03(7) | 0.93 ± 0.06 (7) | 0.91 ± 0.04 (6) | 1.00 ± 0.12 (7) |  | 0.176 | 0.678 | 0.448 | 0.51 | 0.265 | 0.612 |  |  |
| *Glut3* | 0.79 ± 0.04 (7) | 0.86 ± 0.12(7) | 0.89 ± 0.12 (6) | 0.93 ± 0.12 (7) |  | 0.623 | 0.438 | 0.275 | 0.605 | 0.014 | 0.906 |  |  |
| *Glut4* | 0.77 ± 0.08 (7) | 0.75 ± 0.12 (7) | 1.06 ± 0.14 (6) | 0.86 ± 0.17 (7) |  | 2.275 | 0.145 | 0.703 | 0.411 | 0.464 | 0.503 |  |  |
| *Insr* | 0.84 ± 0.05 (7) | 0.91 ± 0.10 (7) | 1.01 ± 0.16 (6) | 1.05 ± 0.13 (7) |  | 1.747 | 0.199 | 0.274 | 0.606 | 0.025 | 0.876 |  |  |
| *Irs1* | 0.91 ± 0.04 (7) | 0.94 ± 0.08 (7) | 0.90 ± 0.06 (6) | 1.03 ± 0.12 (7) |  | 0.249 | 0.623 | 0.959 | 0.338 | 0.433 | 0.517 |  |  |
| *Igf1* | 0.81 ± 0.03 (7) | 0.85 ± 0.08 (7) | 1.12 ± 0.06 (6) | 1.06 ± 0.12 (7) |  | 9.2 | **0.006** | 0.012 | 0.914 | 0.329 | 0.572 |  |  |
| *Gsk3*b | 0.87 ± 0.04 (7) | 0.90 ± 0.07 (7) | 0.99 ± 0.13 (6) | 0.98 ± 0.12 (7) |  | 1.17 | 0.291 | 0.002 | 0.963 | 0.035 | 0.853 |  |  |
| *Pik3*ca | 0.85 ± 0.04 (7) | 0.89 ± 0.07 (7) | 0.94 ± 0.11 (6) | 1.04 ± 0.13 (7) |  | 1.544 | 0.226 | 0.59 | 0.45 | 0.14 | 0.712 |  |  |
| *Rps6kb1* | 0.62 ± 0.02 (7) | 1.33 ± 0.09 (7) | 1.41 ± 0.15 (6) | 0.79 ± 0.06 (6) |  | 2.138 | 0.158 | 0.264 | 0.612 | 54.95 | **<0.0001** | **VEH/SAL vs. VEH/LIR p<0.0001** | **STZ/SAL vs. STZ/LIR p=0.0068** |
|  |  |  |  |  |  |  |  |  |  |  |  | **VEH/SAL vs. STZ/SAL p<0.0001** | **VEH/LIR vs. STZ/LIR p= 0.0161** |
| *Mtor* | 0.76 ± 0.06 (7) | 0.81 ± 0.08 (7) | 0.96 ± 0.17 (6) | 1.02 ± 0.16 (7) |  | 2.605 | 0.12 | 0.174 | 0.68 | 0.002 | 0.962 |  |  |
| *Cd68* | 0.85 ± 0.12 (8) | 0.85 ± 0.07 (8) | 2.34 ± 0.51 (6) | 1.73 ± 0.25 (7) |  | 21.845 | **< 0.0001** | 1.424 | 0.244 | 1.427 | 0.244 |  |  |
| *Il1b* | 0.64 ± 0.12 (8) | 0.66 ± 0.08 (8) | 1.19 ± 0.22 (6) | 1.02 ± 0.06 (7) |  | 13.423 | **0.001** | 0.358 | 0.555 | 0.61 | 0.442 |  |  |
| *Il6* | 1.11 ± 0.16 (8) | 1.07 ± 0.20 (7) | 0.80 ± 0.08 (6) | 0.90 ± 0.06 (7) |  | 3.854 | 0.061 | 0.065 | 0.801 | 0.317 | 0.579 |  |  |
